# Artificial intelligence in digital pathology diagnosis and analysis: technologies, challenges, and future prospects

**DOI:** 10.1186/s40779-025-00680-6

**Published:** 2026-01-04

**Authors:** Xiu-Ming Zhang, Tian-Hong Gao, Qiu-Yu Cai, Jia-Bin Xia, Yu-Ning Sun, Jian Yang, Wei-Han Li, Sheng-Xu-Ming Zhang, Heng-Rui Lou, Xiao-Tian Yu, Kai-Wen Hu, Jing-Wen Ye, Jin-Xing Zhang, Jie Lei, Le-Chao Cheng, Lin-Jie Xu, Qing Chen, He-Xiang Wang, Mei-Fu Gan, Cheng Lu, Nan Pu, Ming-Li Song, Xin Chen, Wen-Jie Liang, Han Lv, Chao-Qing Xu, Zai-Yi Liu, Jing Zhang, Kai Yan, Zun-Lei Feng

**Affiliations:** 1 Department of Pathology, the First Affiliated Hospital, Zhejiang University School of Medicine, Hangzhou, 310000 China; 2 School of Software Technology, Zhejiang University, Hangzhou, 310000 China; 3 State Key Laboratory of Blockchain and Data Security, Zhejiang University, Hangzhou, 310000 China; 4 Department of Electrical and Computer Engineering, National University of Singapore, Singapore, 119077 Singapore; 5 Department of Interventional Radiology, The First Affiliated Hospital with Nanjing Medical University, Nanjing, 210000 China; 6 College of Computer Science, Zhejiang University of Technology, Hangzhou, 310000 China; 7 School of Computer and Information, Hefei University of Technology, Hefei, 230000 China; 8 Department of Radiology, the Affiliated Hospital of Qingdao University, Qingdao, 266000 Shandong China; 9 Department of Pathology, Taizhou Hospital of Zhejiang Province Affiliated to Wenzhou Medical University, Taizhou, 318000 Zhejiang China; 10 Department of Radiology, Guangdong Provincial People’s Hospital (Guangdong Academy of Medical Sciences), Southern Medical University, Guangzhou, 510000 China; 11 The Department of Information Engineering and Computer Science, University of Trento, 38123 Trento, Italy; 12 School of Computer and Computing Science, Hangzhou City University, Hangzhou, 310000 China; 13 Department of Radiology, School of Medicine, Guangzhou First People’s Hospital, South China University of Technology, Guangzhou, 510000 China; 14 Department of Radiology, the First Affiliated Hospital, College of Medicine, Zhejiang University, Hangzhou, 310000 China; 15 Department of Radiology, Beijing Friendship Hospital, Capital Medical University, Beijing, 100000 China; 16 Department of Neonatology, Children Hospital of Fudan University, Shanghai, 201102 China

**Keywords:** Artificial intelligence (AI), Pathology images, Quantitative feature, Pathology foundation model

## Abstract

Artificial intelligence (AI) offers transformative potential in pathology, where histopathological images remain the diagnostic gold standard due to their rich morphological and molecular information. While the rapid development of AI-driven computational pathology tools is revolutionizing disease interpretation, these technologies have not yet been systematically evaluated. Therefore, this review systematically evaluates AI applications across the diagnostic continuum, from image preprocessing and tumor classification to prognostic stratification and the discovery of predictive biomarkers. It presents a technical taxonomy of the algorithms and foundation models powering these applications, benchmarking their performance across diverse diagnostic tasks through rigorous comparative analyses. It also identifies critical challenges in clinical translation, including computational scaling, noisy annotations, interpretability gaps, and domain shifts. Finally, it proposes a roadmap for advancing AI applications in precision oncology and pathological research. By bridging technological innovation with clinical needs, this review aims to accelerate the integration of robust, unified, scalable AI solutions into diagnostic workflows.

**Supplementary Information:**

The online version contains supplementary material available at 10.1186/s40779-025-00680-6.

## Background

Cancer has become a significant public health concern worldwide. According to the latest Annual Cancer Report for 2024 and Global Cancer Burden Data for 2023 from the International Agency for Research on Cancer, part of the World Health Organization (WHO), there were 19.29 million new cancer cases and 9.96 million cancer-related deaths globally in 2023 [1–3]. The top 3 cancers by incidence rates were lung cancer, prostate cancer, and colorectal cancer (CRC) in men, and breast cancer, lung cancer, and CRC in women. Over recent decades, cancer cases have risen steadily due to population aging and lifestyle changes. Research by the WHO predicts that the annual number of new cancer cases worldwide will increase by 77% by 2050, translating to over 35 million new cases yearly [1]. Early detection and effective intervention can lead to long-term survival for many patients with cancer. Despite significant advancements in medical technology, cancer survival rates remain low. Because of issues such as limited early screening, early diagnosis of cancer, and effective postoperative management, these remain global challenges. Therefore, there is an urgent need to find more effective methods for cancer diagnosis and treatment.

Pathology image analysis is considered the gold standard in the clinical diagnosis of tumors. Pathologists determine the tumor’s type, degree of differentiation, and grade by observing cell and tissue morphology in pathology images, providing crucial prognostic information to support clinical decision-making. The techniques, such as immunohistochemistry (IHC) and genetic testing, can assist in guiding personalized clinical treatments, including targeted therapy and immunotherapy [4–6]. With the development of high-definition pathology image scanning systems, pathology has transitioned from the traditional microscope era to the digital era.

A key feature of digital pathology is its whole-slide imaging capability, which enables the complete digitization of physical slides into virtual slides using high-resolution scanners. This digitization enables pathology images to be interactively explored and seamlessly zoomed, while also supporting multi-focal plane microscopy [7]. Digital pathology, characterized by whole-slide images (WSIs), not only preserves the diagnostic integrity of traditional microscopy but also forms the foundation for remote consultations, pathology education, and image sharing and archiving, fundamentally transforming how pathologists interact with histological data. A digital pathology system typically comprises scanning hardware, storage infrastructure, image management platforms, and viewing software, all of which can be seamlessly integrated with hospital information systems and electronic medical records, greatly enhancing workflow efficiency and image reusability. The core characteristics of digital pathology can be summarized as high-fidelity image reproduction, remote accessibility, intelligent analytical capability, and system-level integration. These features are collectively driving pathology toward higher accuracy, reproducibility, and standardization, providing a solid foundation for the realization of precision medicine [8, 9].

The availability of large-scale digital pathology datasets has enabled the application of artificial intelligence (AI) techniques, including machine learning (ML) and deep learning (DL), in various computational pathology tasks [10, 11]. These applications encompass pathology image processing and virtual image generation, tumor screening and diagnosis, prognostic prediction, and biomarker discovery. Recent advances in foundation model architectures such as vision transformers (ViT), convolutional neural networks (CNNs) with self-supervised learning frameworks (e.g., MoCo, SimCLR), and emerging multimodal encoders have facilitated the development of AI-powered diagnostic systems that integrate multimodal data sources, including clinical records, radiomic features, genomic profiles, and proteomic data [12–14]. Leveraging self-supervised learning paradigms, these models can extract meaningful patterns from vast unlabeled datasets, enabling generalization across multiple organ systems without requiring extensive manual annotations [15, 16]. Such AI-powered diagnostic systems represent standardized, reproducible analytical tools that augment pathological practice by enhancing the efficiency and accuracy of tumor screening, diagnosis, prognostic prediction, and therapeutic decision-making [17, 18]. While current implementations remain adjunctive to clinical decision-making and cannot supersede expert pathological assessment, they demonstrate measurable potential to mitigate diagnostic variability and enhance operational efficiency in pathology practice [19, 20]. This technological evolution is poised to transform intelligent pathology systems; however, rigorous clinical validation and compliance with regulatory requirements remain prerequisites for their full integration and adoption.

This review first introduces the digital pathology acquisition pipeline and major AI applications in clinical digital pathology, including data preprocessing and generation, tumor screening and diagnosis, prognostic prediction, and biomarker discovery, to provide a clear overview of how AI techniques are integrated into clinical workflows. It then systematically reviews the technical methodologies used in these applications, analyzing their use cases, advantages, and limitations to summarize effective practices and remaining challenges. Furthermore, it surveys state-of-the-art foundation models for pathology image analysis, detailing their architectures and task coverage, and includes comparative experiments to help researchers choose appropriate models. Unlike previous reviews, this work integrates traditional ML models, modern DL methods, and emerging foundation models into a unified and up-to-date perspective, showing the evolution of AI methodologies. Finally, it catalogs publicly available datasets, discusses key challenges, and outlines future research directions for intelligent pathology diagnosis to promote innovation and clinical translation.

## Sample preparation, image acquisition, and computational processing in digital pathology

**Fig. 1:**
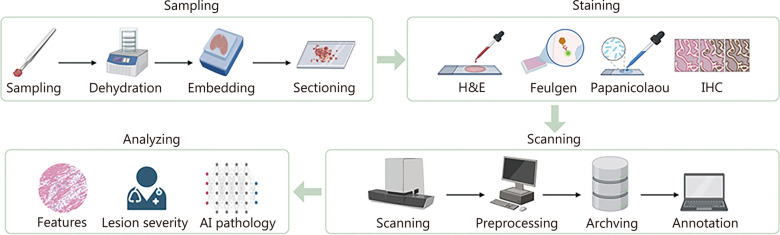
Introduction to the digitized pathology image formation process. H&E hematoxylin and eosin, IHC immunohistochemistry, AI artificial intelligence

In digital pathology, pathology images of existing tissue samples must first be acquired. The transition from a physical tissue sample to a computationally analyzable digital image in pathology involves a multi-stage workflow, encompassing sample preparation, staining, digitization, and computational enhancement. The whole digitized pathology image formation process includes sample preparation and staining, slide digitization and quality control, computational processing, and image analysis and diagnosis, which are described in the following section (Fig. 1).

### Sample preparation and staining

Pathological examinations primarily focus on histology (tissues) and cytology (cells). For histological analysis, tissue samples are fixed, dehydrated, embedded in paraffin, and sectioned into thin slices (typically 4–5 μm) for mounting on glass slides. Specific tissues, such as bone, may require pre-treatment like decalcification. Cytological samples are collected from sources like body fluids or fine-needle aspirations, followed by smearing and fixation [21].

Staining is critical for visualizing cellular structures. Hematoxylin and eosin (H&E) stain is the most common stain, rendering nuclei blue-purple and cytoplasm pink, and is used for basic histomorphological analysis. Specialized stains are employed for specific purposes: IHC for localizing specific protein biomarkers, multiplex immunofluorescence (mIF) for analyzing multi-target co-expression and spatial profiling of microenvironments, and others like Feulgen for DNA quantification [22–24].

### Slide digitization and quality control

Following staining, glass slides are scanned using whole-slide scanners to generate high-resolution digital images (often exceeding 100,000 pixels in width and height). This process incorporates quality control to identify and mitigate artifacts (e.g., blurring, focus issues) [25]. Subsequently, preprocessing steps, such as denoising, color correction, and brightness adjustment, are applied to enhance image clarity [26]. The resulting images are archived in multi-resolution formats to facilitate examination at various magnifications and may be annotated by pathologists.

### Computational processing: stain normalization and image transformation

Computational preprocessing is essential to ensure consistency and enable robust analysis, particularly for AI applications. These techniques algorithmically standardize the color and style of all images to a common reference, effectively removing non-biological noise. This process is indispensable for creating a homogeneous dataset, thereby enhancing the accuracy, reliability, and overall performance of downstream computational analyses and AI applications.

#### Staining normalization

H&E staining coloration varies due to factors like staining protocol, dye batch, and scanning conditions. Stain normalization mitigates this variability to standardize image appearance for computational analysis. Traditional methods include: 1) histogram transformation [27], which maps the color distribution of an image to a reference; 2) separated transformation [28], which applies distinct transformations to individual pixels, though this can introduce artifacts and is computationally intensive; 3) unified transformation [29], which decomposes pixels into stain components and applies a single transformation, offering improved consistency [30–32]. DL architectures, including generative adversarial networks (GANs) [33, 34], transformers [35], and diffusion models [36], have been increasingly adopted for more effective and robust stain normalization [37, 38].

#### Pathology image transformation

Advanced AI techniques now enable the cross-modal transformation of pathology images. For instance, DL models can generate virtual IHC or mIF images directly from H&E-stained images [39, 40]. These methods, primarily based on variational autoencoders [41], GANs [42–44], and diffusion models [36], hold promise for reducing staining costs and accelerating the production of multimodal data [45, 46]. However, significant challenges remain, including the generation of uncontrollable artifacts, limited generalizability across domains, and unverified quantitative reliability. The lack of standardized clinical validation and pathologist acceptance currently restricts their use primarily to auxiliary research and screening, not primary diagnosis.

### Image analysis and diagnosis

The final step involves the examination of digitized images by pathologists, who assess cellular morphology and tissue architecture to render a diagnosis. This process is increasingly supported by AI and ML, which enable automated analysis and are becoming integral to modern diagnostic workflows [47, 48].

## AI applications in digital pathology

**Fig. 2:**
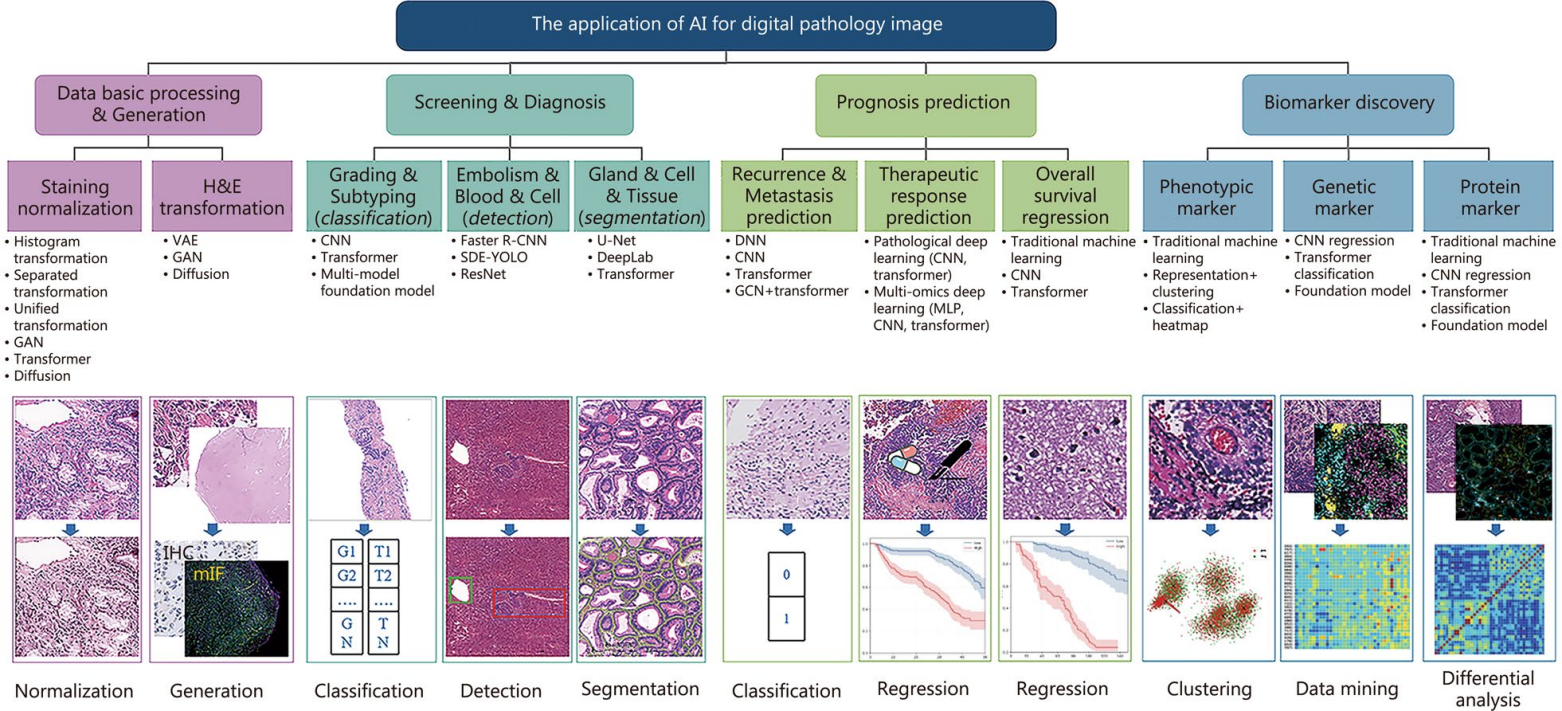
AI applications in digital pathology primarily encompass basic pathology image processing, clinical screening and diagnosis, prognosis prediction, and biomarker discovery. The associated computational tasks and corresponding AI techniques are systematically summarized for each application. H&E hematoxylin and eosin, GANs generative adversarial networks, CNN convolutional neural network, VAE variational auto-encoders, ResNet residual network, U-Net U-shaped network, DNN deep neural network, GCN graph convolutional network

Following the acquisition of digital pathology images, AI applications are employed for multiple tasks, including image processing and virtual image generation, tumor screening and diagnosis, prognostic prediction, and biomarker discovery [49–54]. These tasks, along with their corresponding features [53, 55–134] in intelligent digital pathology analysis, are summarized in Table 1. Overall, the application of AI in this field encompasses a range of areas, from basic image processing [27–29, 33–36] to clinical diagnosis [49–54, 135–140], prognosis prediction [51, 52, 110–112, 129, 133, 141–150], and biomarker discovery [98, 99, 101–103, 149–154] (Fig. 2).

**Table 1:** Summary of the tasks and corresponding features in intelligent digital pathology analysis

Types	Analysis tasks	Pathological features	Computer features	Methods	References
Tumor internal feature analysis	Histological typing; Pathological grading; Necrosis analysis; Vascular and neural invasion; Lymph node metastasis	Cellular morphology, tissue structure, and staining characteristics; Morphological characteristics of tumor cells, tissue structure, and cell differentiation; Tissue structure, cell disintegration, and staining characteristics; Tumor cells invade blood vessels, lymphatic vessels, and nerves; Tumor cell heterogeneity, morphological changes	Haralick texture features, Riesz features, and DL features; Multiscale handcrafted features, DL features; GLCM features, DL features; DL features; Tumor microenvironment features, DL features	SVM, RF, naive Bayes, CNN; SVM, RF, k-nearest neighbors, CNN, GCN, MIL; SVM, CNN; CNN, MIL; Logistic regression, AdaBoost, CNN, MIL	[55–87]
Tumor microenvironment feature analysis	Tumor-stroma ratio; Tumor-infiltrating; Lymphocytes; Stromal Maturity	Cellular morphology, texture; Lymphocytes infiltrating tumor tissue; Maturity of tissue surrounding the tumor	Wavelet features, DL features; Graph features, DL features; DL features	Watershed algorithm, CNN, U-Net, AdaBoost, decision tree; Graph algorithms, CNN, MIL; CNN, RF	[83, 84, 88–93]
Tumor biomarker expression prediction	Immunohistochemistrical biomarkers; Molecular biomarkers	Protein expression levels; Gene expression and metabolism; Characteristics of tumor cells	DL features	SVM, LDA; CNN	[53, 92, 94–109]
Tumor prognosis analysis	Recurrence prediction; Metastasis prediction; Overall survival; Prediction	Tumor size, grading, and molecular characteristics; The interaction between the tumor’s physical properties and microenvironment; Health status and treatment response	Quantitative features; CGA features, TABS features; DL features	LDA, SVM; RF, SVM; Cox proportional hazards model, k-nearest neighbors, SVM; RF, CNN, transformer	[105, 110–124]
Tumor treatment efficacy analysis	Neoadjuvant therapy; Targeted therapy; Immunotherapy	Tumor size, morphology, biomarker expression levels, and cell surface proteins	DL markers	CNN, transformer	[125–132]
Tumor biomarker discovery	Prognostic; Biomarkers; Therapeutic; Biomarkers	Cellular and histological features	Quantitative cell features and tissue distribution features	Watershed algorithm, CNN, transformer, foundation model	[133, 134]

*SVM* support vector machine, *CNN* convolutional neural network, *GCN* graph convolutional network, *U-Net* U-shaped network, *GLCM* gray-level co-occurrence matrix, *CGA* cellular and glandular architectural, *TABS* tumor plus adjacent benign signature, *LDA* linear discriminant analysis, *RF* random forest, *DL* deep learning, *MIL* multiple instance learning

### Pathology image screening and diagnosis

#### Histological typing

Histological typing is not only crucial for pathology image analysis but also directly related to the choice of treatment plan and prognosis assessment. It is well-known that different histological types of cancers may exhibit vastly different biological behaviors and therapeutic responses, even when originating from the same organ. Therefore, accurate histological typing is essential for devising personalized treatment plans. In clinical practice, pathologists classify tumors into specific histological subtypes based on cell morphologies, tissue structures, and H&E staining characteristics, combined with their prior knowledge. In AI-powered systems, histological typing can be achieved using traditional ML, DL, or techniques combining quantitative features with deep models. ML methods employ decision trees, naive Bayes (NB), and support vector machines (SVMs) for typing tasks. In contrast, DL methods are based on CNN [49, 50], Transformer [51, 52], and multi-model foundation model [53, 54]. Techniques that integrate quantitative features with DL models utilize shape, color, and texture characteristics, combined with neural network classifiers, to automatically classify tumors [55–64].

#### Pathological grading

Pathological tumor grading is performed by pathologists through the evaluation of cellular morphological features such as cell size, nuclear pleomorphism, and mitotic activity, as well as tissue architectural patterns, with the WHO grading system being widely used for this purpose. In this system, a higher tumor grade generally corresponds to increased malignant potential and a less favorable prognosis, whereas a lower grade suggests more indolent biological behavior and better anticipated outcomes. With the advancement of computational pathology, ML and DL techniques have been increasingly employed to assist and augment the grading process. Conventional ML methods often rely on handcrafted morphological features combined with classifiers such as SVMs or random forests (RFs) [65–67]. In contrast, DL approaches typically leverage CNNs and multiple instance learning (MIL) frameworks to automatically learn discriminative features from WSIs [68, 69]. Furthermore, integrating extracted handcrafted features quantitatively with DL models has shown promise in enhancing both predictive performance and model interpretability, contributing to more reliable computer-aided grading systems [70, 71].

#### Necrosis analysis

Necrosis denotes cell death caused by disease, injury, or ischemia. Morphologically, necrotic areas typically exhibit disrupted tissue architecture, cellular disintegration, pale staining, and a loose structural appearance under microscopic examination. The extent and spatial distribution of necrosis are clinically significant, as they serve as important indicators of tumor aggressiveness, reflecting rapid growth and inadequate vascular supply [72]. Furthermore, necrosis patterns have been consistently associated with treatment response and patient survival outcomes [73]. Therefore, the primary goal of necrosis analysis is to accurately identify and quantify necrotic areas within tumor tissue. From a computational perspective, necrosis analysis is commonly performed using methods that integrate quantitative image features with DL models. These approaches typically extract morphological and textural features from WSIs, which are subsequently fed into ML or DL classifiers to automate the detection and segmentation of necrotic regions [72–75].

#### Vascular and nerve invasion

Vascular and nerve invasion stands for the phenomenon wherein tumor cells infiltrate blood vessels, lymphatic vessels, or peripheral nerves. This pathological feature is frequently observed in various malignant tumors and serves as a critical indicator of tumor aggressiveness, metastatic propensity, and clinical prognosis [76–80]. Vascular invasion may facilitate the dissemination of tumor emboli, thereby elevating the risk of hematogenous metastasis to distant organs [76]. Similarly, lymphatic vessel invasion is closely associated with an increased likelihood of lymph node metastasis (LNM). Perineural or intraneural invasion can lead to clinical symptoms such as localized pain and neurological deficits [77]. Accurate detection of these invasive patterns is therefore essential for informing treatment strategies and predicting patient outcomes. In computational pathology, DL approaches have been increasingly applied to identify vascular and nerve invasion [78, 79]. These methods typically employ detection frameworks such as Faster R-CNN [135] and SDE-YOLO [136] to identify embolisms, blood, and cells for subsequent analysis. For instance, a dual-branch network architecture combining classification and localization branches has been employed to distinguish between invaded and normal vascular structures while simultaneously localizing regions of cancerous infiltration [80].

#### LNM

LNM refers to the spread of tumor cells through the lymphatic system to the lymph nodes. The detection is a critical determinant in cancer staging, significantly influencing both treatment planning and prognostic evaluation. Histologically, LNM may present as heterogeneous tumor cell populations with altered morphology, often forming distinct boundaries against the background of normal lymphoid tissue. DL algorithms have demonstrated considerable efficacy in detecting LNM across various cancer types, including head and neck squamous cell carcinoma (HNSCC) [81], breast [82], bladder [83], colorectum [84] and cervix [85, 86], by integrating quantitative features with deep models to leverage CNN-derived risk features and tumor microenvironment (TME) characteristics such as lymphatic infiltration [87].

#### Tumor-stroma ratio (TSR)

TSR represents the ratio of stromal tissue (supportive tissue, such as connective tissue and blood vessels) to tumor cells within a tumor. A high TSR frequently correlates with an unfavorable prognosis, as the abundant stroma can create a microenvironment that drives tumor invasion and metastasis. In contrast, a low TSR is often indicative of a more favorable prognosis, suggesting a more contained and less invasive tumor. ML and DL play distinct yet complementary roles in TSR analysis [83, 84]. The core strength of ML methods lies in their interpretability, enabling the quantification of specific pathological features and thereby providing clear support for clinical judgment. In contrast, DL techniques excel at automatically processing complex spatial information within the TME. Commonly used segmentation models, such as U-Net [137], DeepLab [138], and Transformer [139, 140], are adopted to segment glands, tissues, and cells for TME analysis. This capability enables tasks such as precisely quantifying immune cell infiltration in the stromal areas of colorectal cancer (CRC), which is crucial for assessing immune responses [88].

#### Tumor-infiltrating lymphocytes (TILs)

TILs are lymphocytes that have entered the tumor tissue, including T cells, B cells, and natural killer cells. The presence is generally considered a manifestation of the body’s immune response against the tumor. Critically, a higher abundance and greater functional activity of TILs correlate with improved response to immunotherapy and more favorable patient outcomes, a relationship notably observed in melanoma and breast cancer. The integration of ML and DL has revolutionized the analysis of TILs [89, 90]. Computational algorithms now quantify TILs density and spatial arrangement to create prognostic biomarkers, while DL models automatically segment tumor and immune regions for downstream analysis [91]. These approaches not only improve reproducibility but also uncover novel quantitative spatial features, providing insights into the tumor immune microenvironment that extend beyond conventional methods.

#### Stroma maturity

Stroma maturity reflects the developmental and functional state of the supportive tissue surrounding the tumor. Mature stroma typically indicates a stable microenvironment that helps inhibit tumor growth and spread, whereas immature stroma may promote tumor progression. Consequently, the evaluation of stroma maturity provides valuable prognostic insights and is increasingly performed using techniques that integrate quantitative image features with DL models. For example, CNNs can be employed to extract high-dimensional features from tumor-associated stroma regions, which can then be processed by RF classifiers to assist in tumor classification tasks [92]. These AI-driven methods enable objective and standardized stromal quantification, potentially uncovering novel biomarkers of the TME [93, 94]. It will enhance analytical consistency and may refine prognostic assessments by detecting subtle morphological patterns that are challenging to evaluate manually.

### Prediction of tumor prognosis

#### Prediction of recurrence

This assessment involves predicting the risk of tumor recurrence following treatment by integrating clinical data with pathology image analysis, encompassing key medical indicators such as tumor size, grade, and molecular characteristics. Understanding these factors can help identify patients who may require more aggressive follow-up treatment or more frequent monitoring. The integration of classifiers, including quadratic discriminant analysis (QDA), linear discriminant analysis (LDA), and SVMs, enables the transformation of prognostic features into robust recurrence risk prediction models. Subsequent research applying these approaches has established that computationally derived morphological models achieve effective stratification of patients based on recurrence risk. For instance, in early-stage non-small cell lung cancer (NSCLC) recurrence prediction, an AI-based morphometric approach has been used to systematically quantify integrated histological parameters, covering nuclear morphology, spatial relationships, nuclear arrangement, shape descriptors, orientation entropy, and texture [125, 126]. These findings validate the significant potential of multi-feature morphometric analysis combined with ensemble classification strategies in enhancing the accuracy of tumor recurrence prognosis.

#### Prediction of metastasis

This assessment focuses on predicting whether the tumor will spread from its original site to other parts of the body. Key determinants include not only histopathological characteristics, such as invasive morphology and tumor boundary integrity, but also dynamic interactions with the TME, particularly angiogenic activity. Accurate metastasis prediction is essential for determining the necessity of systemic treatments, such as chemotherapy or targeted therapies. In computational approaches, traditional ML methods, including RFs and SVMs, have been widely applied to model metastasis risk based on quantifiable cellular and structural attributes. Evidence from recent studies suggests that integrating multimodal data, such as histopathological imaging with molecular profiles, thinning such models may improve their predictive robustness [127, 128]. Furthermore, while current models rely largely on handcrafted features, emerging DL models, including DNNs [110], CNNs [129], Transformers [51, 52], and GNNs [141], show promise in autonomously learning discriminative patterns associated with metastatic propensity, potentially enabling more generalizable and precise risk stratification.

#### Prediction of therapeutic efficacy

The prediction of therapeutic efficacy mainly involves tasks such as predicting the efficacy of neoadjuvant therapy, targeted therapy, and immunotherapy. These tasks rely on a deep understanding and precise quantification of tumor tissue characteristics. For example, in neoadjuvant therapy, analyzing tumor size, morphology, and biomarker expression can predict the responsiveness to chemotherapy, radiotherapy, or combined therapy [129, 130]. In targeted therapy and immunotherapy, analyzing tumor gene mutations, cell surface proteins, and the immune microenvironment is key to predicting the efficacy of specific therapies and survival rates. The prediction of therapeutic efficacy relies on medical features that underscore the importance of individualized and dynamic monitoring [131, 132]. Inter-patient variability in pathological characteristics and treatment responses necessitates personalized therapeutic strategies. To address these complexities, researchers have turned to deep learning. They employ models such as CNNs and Transformers [142–148] to automatically extract prognostic features from pathological data and to integrate multi-omics information [143, 144, 148] for response prediction. Additionally, tumor properties may evolve during therapy through mechanisms such as acquired gene mutations or drug resistance, highlighting the critical need for real-time monitoring and adaptive adjustment of treatment regimens to enhance both clinical outcomes and predictive accuracy. This process relies on advanced data analysis and ML techniques to handle and interpret large amounts of clinical data. DL biomarkers based on CNNs and transformers were used to predict the response of patients with advanced unresectable gastric cancer to a programmed cell death 1 inhibitor combined with chemotherapy.

#### Prediction of overall survival

The objective of overall survival prediction is to develop quantitative methods that synthesize tumor pathology, patient health, and treatment response data, thereby enabling the estimation of time from diagnosis to death. Traditional ML methods [133], such as Cox proportional hazards models and random survival forests, have been widely applied in survival analyses. DL methods, with their powerful feature extraction capabilities and ability to model complex nonlinear relationships, also show great potential in survival analysis [111, 112]. The combination of both techniques can further enhance model prediction performance and robustness [113–116].

In recent years, the integration of pathological data with other diagnostic modalities has attracted growing interest for improving patient outcome prediction [111, 117–119]. While unimodal pathology approaches, primarily analyzing histological and cytological characteristics, can yield useful prognostic information, their dependence on a single data type frequently hinders comprehensive representation of disease complexity, leading to constrained predictive accuracy and clinical utility for particular tumor types [120, 121]. This limitation has propelled multi-modal and multi-omics integration into a prominent research focus. By synthesizing data from histopathology, radiology, molecular profiling, and proteomics, these methodologies deliver a more comprehensive perspective on tumor heterogeneity and temporal evolution. Accumulating evidence confirms that multi-modal frameworks consistently outperform unimodal systems in forecasting overall survival, disease recurrence, and treatment efficacy [52, 150]. Moreover, these integrated models facilitate the identification of underlying molecular mechanisms and pathological pathways, thereby accelerating progress toward personalized therapeutic interventions.

### Biomarker discovery

Current tumor biomarkers encompass genetic, protein, histopathological, phenotypic, exosomal, and metabolite markers. Among these, exosomal and metabolite markers represent emerging classes. Most existing AI-assisted methods focus on analyzing markers across the spectrum, from histopathological phenotypes to genetic and protein expressions, with the ultimate goal of uncovering fundamental molecular mechanisms. The comprehensive characterization of these biomarkers facilitates improved diagnostic accuracy, prognostic assessment, and the identification of therapeutic targets in oncology research. Advanced computational methods, particularly AI-driven approaches, are increasingly employed to analyze multimodal biomarker data derived from digital pathology, next-generation sequencing, and mass spectrometry. The synergistic analysis of these complementary biomarker categories enables a more holistic understanding of tumor biology and clinical behavior.

#### Histopathological phenotypic markers

Histopathological phenotypic biomarkers are quantitative indicators with diagnostic and prognostic value, which are identified by analyzing morphological characteristics and spatial distribution patterns in tissue sections. Traditional approaches employ ML methods [151, 152] to extract microscopic features, including nuclear morphology and tissue architecture, and correlate them with clinical outcomes. With advancements in DL techniques, modern models now extract multiscale features encompassing both macroscopic (tissue-level) and microscopic (cellular-level) characteristics for diagnostic and prognostic predictions. In parallel, 2 main approaches have been developed to interpret these features: representation clustering [141, 149] and heatmap visualization [153, 154]. The former involves clustering learned deep representations to identify outcome-associated feature clusters, whereas the latter utilizes model attribution techniques to generate heatmaps highlighting histopathological features relevant to diagnosis or prognosis. A key benefit of DL-derived phenotypic biomarkers lies in their ability to directly capture tumor morphological heterogeneity. Moreover, they present a potentially more accessible alternative to molecular assays in terms of cost and scalability. Current research priorities focus on developing interpretable AI models to identify biologically meaningful phenotypic features, as well as investigating their cross-omics correlations with genomic and proteomic biomarkers.

#### Genetic biomarkers

Tissue-derived tumor biomarkers are remarkably diverse. AI-assisted genetic biomarker detection enables pathologists to rapidly and accurately identify tumor-associated key genes and define the specific genetic biomarkers for tumor characterization. Traditional approaches for discovering tumor gene biomarkers primarily rely on molecular profiling to generate large-scale genomic datasets, followed by cluster analysis to identify tumor-associated genetic markers [94–97]. In comparison, DL [98, 99] and foundation models [153, 154] offer a more streamlined strategy by predicting genetic biomarkers directly from histopathological images. This AI-driven paradigm markedly enhances the efficiency and cost-effectiveness of genetic biomarker screening and prediction [100].

#### Protein markers

IHC is a technique that detects the expression of specific proteins within cells by staining tissue sections with antibodies against them. Traditional ML approaches quantify protein expression by segmenting and statistically analyzing relevant cells to identify tumor-associated protein biomarkers [101]. The field has been significantly advanced by the introduction of DL and foundational models, which enable more sophisticated and direct prediction of protein biomarkers from histopathological images [53, 98, 99, 102, 103]. These AI-driven methods not only automate the quantification process but also capture subtle morphological patterns associated with protein expression that may evade conventional analysis. For instance, DL models can predict protein marker status directly from H&E-stained WSIs, providing a cost-effective and scalable alternative to traditional IHC in certain scenarios [104–106]. Ongoing technological refinements are steadily enhancing the precision and efficiency of protein biomarker discovery. The integration of multi-modal data and the development of interpretable AI frameworks are expected to further elucidate the relationships between protein expression, tumor morphology, and clinical outcomes, ultimately strengthening their role in diagnostic and prognostic assessment.

## Intelligent pathology image analysis technologies

### Traditional ML-based technologies

ML is a field that develops algorithms capable of improving their performance at a given task through exposure to data, typically by optimizing an objective (e.g., loss function or reward) while balancing generalization to unseen data. The main difference between traditional ML and DL is that it relies on manual design of features, simulating or implementing human learning behavior to obtain data features from a large amount of historical data [155].

This section will introduce the various techniques and methods used in intelligent pathology diagnostic analysis, with a particular focus on their applications in tasks such as histological typing, pathological grading, and assessing prognosis. It begins by reviewing the traditional ML methods [55–61, 70, 88, 89, 110, 113–119, 123, 124, 133, 156–161] that established the computational foundation for the field (Table 2), which leverage handcrafted morphological features and a diverse array of classifiers in tasks including WSI classification, patch-based analysis, and regression-based prognosis modeling.

**Table 2:** Summary of traditional ML techniques for digital pathology image analysis

Methods	Executable tasks	Features	Classifiers	References
Whole slide image classification	Histological typing; Pathological grading; Prognostic analysis; LNM	Mass thickness, cell size and shape consistency, adhesion between cancer and normal cells, epithelial cell enlargement, naked nuclei in benign tumors, coarse chromatin, normal nucleolus, and mitotic count; Luminal characteristics (shortest path from nucleus to nearest cavity, epithelial nuclei ratio, average shortest distance to lumen) and architectural characteristics (matrix and nuclear orientation changes); Quantitative histomorphological features of nuclear shape and orientation; Appearance features of LNM	Decision trees (C4.5, bagged, and boosted), least squares regression, KNN, and boosted Bayesian multiresolution; SVM, logistic regression, NB, LDA, and RF; LDA; GBM, RF, and NB	[55–61, 156–159]
Patch classification	Histological typing; Pathological grading	Mass thickness, cell size and shape consistency, adhesion between cancer and normal cells, epithelial cell enlargement, naked nuclei in benign tumors, coarse chromatin, normal nucleolus, and mitotic count; Luminal characteristics (shortest path from nucleus to nearest cavity, epithelial nuclei ratio, average shortest distance to lumen) and architectural characteristics (matrix and nuclear orientation changes)	Decision trees (C4.5, bagged, and boosted), least squares regression, and rotation forest; SVM, logistic regression, NB, and LDA	[55–61, 156, 160]
Segmentation	Histological typing;	Color features of overlapping nuclei	k-means and watershed algorithms	[89, 110, 113–119, 123, 124, 161]
regression analysis	Prognostic analysis; Pathological grading	Co-occurrence gland angle, tumor-adjacent benign features, demographic, clinical, imaging, and surgical data, texture features (from co-occurrence statistics and local binary patterns), structural features (nuclear orientation, texture, shape), feature-driven local cell cluster maps, and TIL spatial features	RF, KNN, logistic regression, SVM, Cox proportional hazards model, minimum redundancy and maximum correlation, QDA, and LDA	
Others	Internal tumor analysis; Vascular and neural invasion; TME analysis; Tumor-infiltrating lymphocytes; Tumor biomarker discovery	Different degrees of malignancy and staining intensity; Appearance characteristics of neurovascular; Characteristics of the tumor-stroma ratio; Density and spatial co-localization characteristics of tumor cells; Pathological labels based on machine learning, tumor-associated benign signature	SVM, GBM, NB, logistic regression; Decision trees, RF, and support; watershed algorithms; Graph algorithm; Watershed algorithms and RF	[55, 70, 88, 89, 133]

*KNN* k-nearest neighbor, *SVM* support vector machine, *NB* naïve Bayes, *LDA* linear discriminant analysis, *RF* random forest, *GBM* gradient boosting machine, *QDA* quadratic discriminant analysis, *LNM* lymph node metastasis, *TIL tumor-infiltrating lymphocytes*

#### WSI classification

WSI classification serves as a fundamental methodology in digital pathology, addressing essential diagnostic tasks including histological typing [156, 160], pathological grading [58], and LNM detection [55]. This approach systematically leverages quantifiable morphological characteristics, such as nuclear morphology, cellular arrangement patterns, and tissue texture features, and processes them through diverse ML classifiers ranging from conventional algorithms to advanced ensemble methods. By integrating domain-specific feature engineering with optimized classification techniques, WSI classification establishes an interpretable and computationally efficient framework for supporting diagnostic decisions across multiple cancer types and clinical scenarios.

*Histological typing task* In the context of histological typing, traditional ML models have been widely applied to histopathology images. The key technical approaches in this area can be summarized as follows: ensemble learning techniques, penalized regression models, and multi-feature classification frameworks.

Several studies [56, 57, 155, 156, 160] have employed ensemble learning strategies to improve classification performance. C4.5, bagged, and boosted decision trees have been applied across 7 public microarray datasets, demonstrating the consistent superiority of ensemble methods over single decision trees [156]. A rotation forest system was developed that projects data into new feature spaces to enhance classifier accuracy and ensemble diversity, showing improved performance over bagging and boosting on breast and prostate cancer datasets, with additional gains from incorporating independent component analysis [160]. In addition to ensemble approaches, some works have focused on specialized classification models. A penalized logistic regression method for multi-class cancer classification achieved comparable or superior performance to SVMs and least squares regression while offering interpretable coefficients and probabilistic outputs [56]. Comparative analyses between NB and KNN classifiers for binary breast cancer classification further established the relative advantage of KNN in this application [57].

Multiple studies explored comprehensive feature extraction and selection approaches for histopathology image analysis. Doyle et al. [157] extracted intensity, texture, architectural, frequency, and orientation features from prostate cancer images and implemented an AdaBoost feature selection method within a boosted Bayesian multiresolution classifier framework. These investigations collectively demonstrate that combining relevant morphological features with appropriately selected classifiers forms an effective strategy for histological typing and grading in computational pathology.

*Pathological grading task* Similarly, cancer grading tasks have increasingly utilized multi-scale feature extraction combined with diverse classifiers to enable automated histological assessment. The main technical approaches in this domain involve integrating architectural, morphological, and domain-specific features with statistical or ensemble classifiers for improved grading performance. Naik et al. [58] developed an automated grading system that integrated low-level pixel information, high-level object features, and domain-specific tissue context using a Bayesian classifier, level-set evolution, and template matching for prostate and breast cancer grading. Glandular and graph-based structural features have been extracted from prostatectomy WSIs and analyzed using LDA for binary classification, demonstrating enhanced Gleason grading capability [158]. Multi-scale nuclear characteristics, including architectural patterns and texture features in breast cancer specimens, have also been analyzed using an RF classifier for tumor grade prediction across different grade categories [159]. Collectively, these studies demonstrate that combining multi-scale histological features, spanning pixel-level, object-level, and tissue-level information, with appropriate classifiers such as Bayesian methods, LDA, or RFs provides an effective framework for automated cancer grading in digital pathology.

*Prognosis analysis task* In the domain of prognostic modeling using handcrafted morphological features, quantitative analysis of nuclear characteristics has emerged as a significant approach for survival stratification. Lu et al. [61] explored the prognostic value of nuclear shape and orientation descriptors in predicting survival outcomes among LN^−^ estrogen receptor (ER)^+^ breast cancer patients. By extracting quantitative features from H&E-stained tumor images and employing cross-validation, their study demonstrated that these morphological features could independently stratify patients into distinct survival groups, highlighting the potential of interpretable nuclear morphology in enhancing prognostic assessment beyond conventional clinical parameters.

*LNM task* In the domain of LNM prediction, comparative evaluation of multiple ML classifiers has emerged as a principal methodological framework. Lu et al. [55] systematically assessed 6 conventional ML models, including decision trees, RFs, SVMs, gradient boosting machines (GBMs), NB, and logistic regression, for predicting LNM status in gastric cancer using internal tumor features. Through tenfold cross-validation, their study demonstrated the superior predictive capability of the GBM model among the evaluated algorithms, underscoring the particular advantage of ensemble-based approaches in LNM classification tasks.

These findings align with broader applications of traditional ML in pathological image analysis, where the strategic combination of engineered features and conventional classifiers has consistently enhanced performance across diagnostic tasks.

For histological typing, ensemble decision trees, penalized logistic regression, and rotation forests have shown strong multi-class cancer prediction capabilities, emphasizing the importance of feature selection and classifier design [56, 57, 155, 156, 160]. In pathological grading, Bayesian classifiers, LDA, and RFs have successfully integrated multi-scale morphological and texture features to improve accuracy [58, 158, 159]. Prognostic investigations have demonstrated that quantitative nuclear morphology and orientation features can effectively stratify patient survival outcomes independent of clinical parameters [61]. Collectively, these studies validate the enduring effectiveness of feature-engineered traditional ML approaches in advancing digital pathology.

#### Patch classification for pathology image analysis

Patch classification serves as an interpretable computational approach in pathology image analysis by integrating handcrafted morphological features with traditional ML classifiers. This methodology primarily addresses 2 key diagnostic tasks: histological typing [59] for cancer subtyping and pathological grading [162] for tumor differentiation. By extracting quantifiable descriptors of cellular and tissue structures, such as nuclear morphology and glandular architecture, and combining them with classifiers ranging from decision trees to SVMs, this approach enables accurate and interpretable tissue analysis. The following sections detail the application of this paradigm to specific diagnostic challenges in cancer classification and grading.

*Histological typing task* Histological typing based on genomic data represents an important extension of traditional ML in cancer computational pathology. Wang et al. [59] systematically explored this direction by employing correlation-based feature selection to identify discriminative genes from microarray data, followed by dimensionality reduction to construct optimized feature sets. Their methodology incorporated multiple classifiers, including decision trees, NB, and SVMs, with model validation performed through leave-one-out cross-validation. This approach demonstrated robust classification performance across different hematologic malignancies, including acute leukemia and diffuse large B-cell lymphoma. Particularly noteworthy was their pioneering work in bridging computational findings with biological interpretation, specifically revealing enzyme-related mechanisms in leukemia pathogenesis.

*Pathological grading task* Pathological grading has witnessed significant advances through the integration of handcrafted morphological descriptors with ML classifiers, establishing an interpretable computational paradigm that captures diagnostically critical tissue patterns. This approach emphasizes feature engineering strategies that quantify both cellular architecture and structural organization.

The methodological foundation is built upon comprehensive feature engineering frameworks that incorporate dual analytical dimensions: luminal characteristics quantifying spatial relationships through nuclear-glandular distance measurements and epithelial nuclear ratios, alongside structural patterns captured via directional filter banks. These engineered feature sets are subsequently processed through subspace reconstruction methodologies to achieve accurate differentiation between low- and high-grade carcinomas [162].

Extending this feature-based paradigm, subsequent methodologies have integrated nuclear segmentation techniques with morphological feature extraction. By implementing SVMs to classify cancer grades based on systematically engineered cellular characteristics, these approaches demonstrate the enduring value of domain-informed feature design in computational grading systems [60].

These graded methodological developments reveal a consistent principle: carefully designed morphological and structural features, when combined with appropriate ML classifiers, provide both robust performance and maintained interpretability. The continued evolution of feature engineering strategies, from basic morphological measurements to sophisticated spatial relationship quantification, has established a solid foundation for objective cancer grading that balances analytical precision with pathological relevance.

#### Regression-based pathology image analysis

Regression-based ML approaches have been widely employed in prognostic analysis within computational pathology, integrating histomorphometric, spatial, and clinical features with a variety of classical classifiers to predict survival, recurrence, and risk stratification across multiple cancer types. Glandular co-occurrence and tumor-adjacent benign signatures combined with RF classifiers have been used for prostate cancer outcome prediction [116, 117]. Supervised learning models, including KNN, RF, SVM, and NB, have been applied to predict biochemical recurrence, demonstrating advantages over conventional statistical approaches [113, 114]. Multiresolution KNN has been utilized for neuroblastoma prognosis [163], and nuclear features combined with Delaunay triangulation have been applied for gland segmentation in prostate cancer studies [164]. Multiple additional investigations [89, 115, 118, 124, 161, 165–168] have further established that graph-based, tissue morphological, and nuclear-glandular features combined with classifiers such as RF, LDA, QDA, logistic regression, and SVM effectively capture prognostic patterns across diverse malignancies.

Collectively, these studies validate regression-based ML as a powerful paradigm for prognostic modeling in computational pathology, demonstrating that multi-scale feature integration with traditional classifiers provides robust predictive capability for cancer outcomes.

#### Other pathology image analysis tasks

Beyond core diagnostic tasks, ML methodologies have demonstrated remarkable versatility across 3 expanding domains of computational pathology: internal tumor characterization, TME investigation, and biomarker discovery. These applications highlight the enduring value of traditional ML approaches in extracting multidimensional clinical insights from pathological images.

Internal tumor characterization leverages multiple analytical strategies to decode tumor architectural complexity. Appearance-based SVMs have been developed to systematically identify necrotic regions in WSIs, providing a quantitative assessment of tumor viability. Complementing this approach, comprehensive comparative analyses of 6 traditional ML models, including decision trees, RFs, and GBMs, have established robust frameworks for predicting multiple invasion patterns and metastatic potential [55, 70].

TME investigation employs computational geometry and spatial analytics to decode cellular ecosystems. Watershed-based segmentation algorithms quantify TSRs and evaluate their prognostic significance, while graph-based algorithms map spatial distributions of TILs within tissue architectures, revealing clinically relevant organizational patterns [88, 89].

Biomarker discovery represents the translational frontier, where pathomics signatures constructed from watershed-segmented features identify novel prognostic markers. Advanced signatures integrating nuclear morphological and architectural features from multiple tissue regions further enable recurrence prediction through RF classification, creating powerful prognostic tools that capture tumor heterogeneity [117, 133].

Collectively, these methodological advances demonstrate that traditional ML approaches, when guided by domain expertise, continue to provide indispensable capabilities for analyzing complex pathological features. By bridging quantitative image analysis with clinical interpretation, these techniques significantly contribute to prognostic assessment and the advancement of precision oncology, establishing a solid foundation for the ongoing integration of computational methods into pathological practice.

### DL-based and quantitative feature-enhanced models for pathology image analysis

**Fig. 3:**
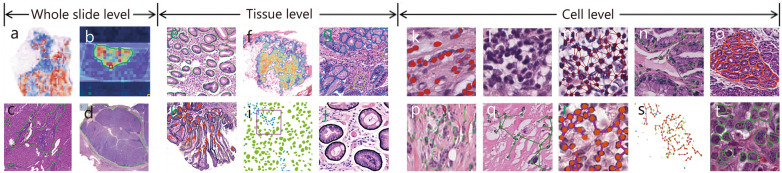
Visualization of quantitative features at the whole-slide, tissue, and cell levels. The slide level includes percentage of cancerous areas (**a**), morphology of cancerous areas (**b**), TIL cluster density and structure (**c**), and tumor morphology and area, using computer-aided analysis to confirm the width, manual area, digital area, and morphological features of the primary tumor (**d**). The tissue level includes gland distribution and morphology (**e**), TME (**f**), tissue morphology and relationships (**g**), gland angles (**h**), local cellular interactions (**i**), and tissue abundance (**j**). The cell level includes nuclear structure and texture (**k**), intensity statistics and co-occurrence (**l**), nuclear morphology and topology (**m**), nuclear morphology and arrangement (**n**), quantitative nuclear features (**o**), nuclear shape and structure (**p**), using DL models to extract nuclear features followed by quantitative methods to extract features related to nuclear shape and structure; nuclear shape, texture, and orientation (**q**), nuclear cluster spatial map features (**r**), spatial distribution and topological features of cell clusters (**s**), and the size, shape, and texture features of cells (**t**). TIL tumor-infiltrating lymphocyte, TME tumor microenvironment

This section focuses on the tasks conducted in intelligent digital pathology technologies based on DL and the combination of quantitative features with deep models, such as WSI classification, patch classification, detection, segmentation, and regression. Corresponding to these tasks, the prevalent DL-based models for pathology image analysis [62–69, 71–84, 86, 87, 90–94, 97, 100, 104–106, 109–112, 119, 120, 123, 129, 130, 169–183] are summarized in this section (Table 3). All of the models depicted are based on either CNN or transformer architectures, each with its unique application scenarios. For example, the ResNet, GoogLeNet, MobileNetV2, convolutional-like vision transformer (ConViT), and graph convolutional network (GCN) are primarily employed for WSI and patch classification tasks; the Faster region-based CNN (R-CNN) and U-shaped network (U-Net) are employed mainly for detection and image segmentation, respectively; and the transformer is mainly employed for regression tasks in prognostic analysis. These computational approaches enable a comprehensive visual analysis across different biological scales. The visualization of quantitative features spans macro (whole-slide), meso (tissue), and micro (cellular) scales, systematically characterizing multiscale information ranging from tumor regions and tissue architecture to nuclear morphology and spatial arrangement [65, 68, 83, 89, 108, 122, 124, 157–159, 161, 165–168, 177, 184] (Fig. 3).

**Table 3:** Summary of the DL models employed for digital pathology image analysis

Methods	Adaptation task	Model architecture	Architecture type	References
Whole slide image classification	Histological typing; Pathological grading; Vascular and nerve invasion; LNM; IHC; Prognosis analysis	Inception V3, GoogLeNet, AlexNet, ResNet; CNN, inception V3, ResNet; LoopNet, ResNet, ConViT; GoogLeNet, ResNet, MobileNetV2; HBNet, ResNet, MFD-Net, MRF-ANN, CNN; VGG, Elastic Net	CNN, transformer	[62–64, 75–77, 80–83, 94, 119, 169–178]
Patch classification	Histological typing; Pathological grading; Vascular and nerve invasion; LNM	CNN, elastic net; ResNet, GCN-MIL, GasMIL, CNN; MVI-DL; VGG, LNMNet, ResNet	CNN, GCN	[65–68, 78, 79, 86, 87, 109, 179–182]
Detection	Vascular and nerve invasion; LNM; IHC	CNN; Faster RCNN, DenseNet; U-Net	CNN, faster R-CNN, U-Net	[79, 87, 109]
Segmentation	Pathological grading; IHC	Hover-Net; U-Net, DeepLIIF, UNet-MobileNet, ResNet	CNN, U-Net	[69, 92, 97, 100, 104]
Regression	Prognosis analysis	DeepConvSurv, DNN, CNN, MobileNetV2, ResNet, swin transformer V2, VGG, LSTM, U-Net	CNN, transformer, LSTM, U-Net	[110–112, 120, 123, 129, 130, 183]
Others	Necrosis analysis; Tumor-stroma ratio; Tumor-infiltrating lymphocytes; Stromal maturity; Molecular biomarker prediction	CNN, DenseNet; VGG; WeakSTIL, ResNet; U-Net, VGG; ResNet	CNN, U-Net	[71–74, 84, 90–93, 105, 106]

*CNN* convolutional neural network, *GCN* graph convolutional network, *GoogLeNet* Google’s inception network, *U-Net* U-shaped network, *LSTM* long short-term memory, *MIL* multiple instance learning, *R-CNN* region-based CNN, *ResNet* residual network, *ConViT* convolutional-like vision transformer, *LNM* lymph node metastasis, *IHC* immunohistochemistry

#### WSI classification

WSI classification in pathology encompasses various diagnostic tasks, including histological subtyping, pathological grading, lymphovascular invasion (LVI) assessment, LNM detection, IHC analysis, and survival prediction. These applications are predominantly implemented through fully supervised learning approaches utilizing CNN architectures and their enhanced variants, with emerging integration of quantitative feature-enhanced methodologies.

*Histological typing task* Histological typing for various cancers has been implemented using diverse CNN architectures such as Inception v3, GoogLeNet, ResNet-50, AlexNet-BC, MSSE-ResNet, and an embedded fusion mutual learning method, often incorporating transfer learning, two-stage architectures, and attention mechanisms to enhance classification performance across different magnifications and datasets. Standard CNN architectures have demonstrated strong performance in basic classification tasks. Inception v3 has been applied to differentiate lung adenocarcinoma, squamous cell carcinoma, and normal tissue from WSIs [169], while GoogLeNet with transfer learning has shown effectiveness on the BreaKHis dataset [170]. The ResNet-50 architecture operating under weakly supervised frameworks has achieved accurate classification without requiring detailed annotations [171]. Methodological enhancements have further advanced the field. AlexNet-BC was specifically designed to address overfitting challenges in pathological image analysis [172]. Attention mechanisms have been incorporated through frameworks like msSE-ResNet, which employs multiscale channel recalibration for classification across magnification levels [173]. Integrated approaches combining DL with traditional ML in two-stage systems have enabled multi-class pathology classification [174]. The embedded fusion mutual learning method represents a significant advancement, demonstrating robust and magnification-independent performance across multiple benchmark datasets [175]. Collectively, these developments confirm the effectiveness of DL-based models in histological typing while highlighting the need for improved interpretability and clinical robustness.

*Pathological grading task* DL approaches for pathological grading primarily utilize multi-scale CNNs, specialized risk stratification models, and ensemble architectures to perform Gleason grading, cancer risk subgroup classification, and label-free glioma grading. At the architectural level, systems integrating multiple CNNs of varying sizes with multiscale decision fusion and data augmentation have demonstrated strong performance in prostate cancer Gleason grading [62]. For specialized risk stratification, the DeepGrade model represents a significant advancement by enabling refined prognostic classification, particularly for intermediate-risk breast cancer cases [63]. Meanwhile, ensemble-based methodologies employing multiple ResNet-18 networks as component classifiers have shown notable success in label-free glioma grading, effectively discriminating between Grade II and III tumors [64]. Current evidence collectively indicates that these sophisticated DL approaches can achieve performance levels comparable to human experts.

*LVI task* LVI serves as the universal pathological term for evaluating vascular invasion across all cancer types, while microvascular invasion (MVI) represents a specialized term specifically used in hepatocellular carcinoma (HCC) assessment. Recent advances in LVI/MVI detection have been driven primarily by multi-task learning and hybrid multi-model frameworks. The first approach employs a dual-branch architecture integrating classification and cell localization for MVI identification in liver cancer [75, 76]. The second adopts a multi-model framework combining classification and detection models for LVI prediction in gastric cancer [77]. These methodological advances highlight the considerable potential of DL in LVI/MVI assessment, while underscoring the need for validation across larger and more diverse patient cohorts to facilitate clinical translation. These methodological advances highlight the considerable potential of DL in LVI/MVI assessment, while underscoring the need for validation across larger and more diverse patient cohorts to facilitate clinical translation.

*LNM task* Current methodologies for LNM detection leverage 3 distinct computational paradigms: competitive benchmarking against human experts, multi-stage diagnostic architectures, and knowledge-transfer enhanced segmentation systems. The CAMELYON16 challenge established key comparative benchmarks by pitting DL models against pathologists in detecting breast cancer metastases [80]. For multi-stage diagnostic frameworks, researchers have developed a two-stage automated system that achieves comprehensive metastasis identification in HNSCC [81]. Meanwhile, the expert-experience embedded knowledge transfer learning (EEKT) model represents another advancement through its integration of automated segmentation with quantitative analysis of lymph-blood vessel invasion features, specifically in breast cancer [82]. These methodological advances demonstrate DL’s capacity to enhance diagnostic accuracy and provide quantitative pathological assessments.

*IHC task* Advanced computational methods are enhancing IHC analysis through 3 primary approaches: automated scoring systems, virtual staining techniques, and multi-modal classification frameworks. Recent advances include an automated IHC scoring system that integrates uncertainty quantification for large-scale proteomic studies [94]. Another innovation employs a multi-scale classification framework enhanced with dual attention mechanisms to support IHC-guided therapeutic decisions [96]. Beyond scoring and classification, a virtual staining approach now enables direct biomarker detection from conventional histochemical specimens [176]. These methodologies demonstrate progressive maturation of computational IHC applications, transitioning from basic automation to sophisticated analytical capabilities. Future development should emphasize the creation of standardized benchmarking datasets and implementation guidelines to address inter-laboratory variability and ensure reproducible performance across diverse clinical environments.

*Survival prediction task* DL approaches for survival prediction have evolved from single-modal image analysis to integrated multi-modal frameworks that combine histopathological features with clinical and molecular data for enhanced prognostic accuracy. Recent advances include a self-calibration architecture with global and detailed branches, enabling simultaneous survival prediction and phenotypic biomarker discovery [106]. Other developments feature multi-stain integration and cross-modal fusion systems that combine histopathology with clinical and molecular data to forecast treatment response [110, 111]. Several methodologies now extract prognostic information directly from WSIs, including multi-scale texture analysis for feature extraction and CNN-based transfer learning frameworks applied to H&E-stained specimens [149, 152, 177, 181]. Additionally, dedicated DL platforms have been created specifically for biomarker discovery in cancer prognosis [133]. These methodologies demonstrate the progressive integration of multiple data modalities and the discovery of novel digital biomarkers directly from histopathological images. Future development should focus on establishing standardized protocols for multi-modal data fusion and validating these approaches in prospective clinical trials to demonstrate their utility in personalized treatment planning and patient stratification.

*Quantitative feature-enhanced models* Hybrid methodologies combining DL with handcrafted quantitative features have emerged as a powerful paradigm for enhancing WSI analysis through feature-level integration and ensemble modeling techniques. Several innovative frameworks illustrate this trend. One approach integrates CNN-extracted risk features with TME characteristics through an AdaBoost classifier for metastasis assessment [83]. Another employs artificial neural networks to combine cell color features with ER^+^ cell proportion data for automated ER scoring [107, 185]. In computational pathology, a cascaded pipeline first performs DL-based nucleus segmentation, then applies traditional classifiers for molecular risk categorization [108]. Beyond diagnostic applications, morphological features describing cellular and nuclear architecture have been integrated with Cox proportional hazards modeling to build prognostic systems for survival prediction [119]. These hybrid approaches demonstrate the synergistic potential of combining data-driven deep features with domain-knowledge-inspired quantitative features.

WSI classification has demonstrated remarkable progress across multiple diagnostic domains, including histological subtyping, pathological grading, LVI/MVI assessment, LNM detection, IHC analysis, and survival prediction. These advances are primarily built upon sophisticated CNN architectures and their enhanced variants, with emerging integration of quantitative feature-enhanced methodologies. The field has evolved from basic classification tasks to comprehensive diagnostic systems capable of providing quantitative assessments and prognostic insights. The next frontier lies in developing unified diagnostic frameworks that seamlessly integrate multi-scale pathological features with clinical context to bridge the gap between computational precision and clinical applicability across all WSI classification tasks.

#### Patch classification for pathology image analysis

Patch classification in computational pathology encompasses multiple diagnostic tasks, including histological typing, cancer grading, MVI detection, and LNM prediction. These applications are predominantly addressed through weakly supervised MIL frameworks that leverage CNNs, GCNs, and attention-based architectures for feature representation. Recent advancements have evolved toward integrated approaches combining domain-specific quantitative features with data-driven DL representations, enabling more precise and generalizable diagnostic capabilities across diverse cancer types while significantly reducing dependency on detailed annotations.

*Histological typing task* AI-powered pathological image analysis has evolved from basic patch-level classification to sophisticated frameworks incorporating self-attention mechanisms, noise-resistant architectures, and large-scale foundation models for enhanced diagnostic accuracy and generalizability across cancer types. Initial research demonstrated the value of combining patch-level CNNs with supervised decision integration for cancer classification [179]. Subsequent innovations include a self-reform framework that dynamically identifies diagnostically critical regions to accelerate WSI analysis [186]. To address data quality challenges, noise-resistant architectures have been developed with enhanced robustness against label noise in diagnostic applications [187]. More recently, the field has seen the emergence of pathology foundation models pre-trained on extensive WSI datasets, enabling versatile diagnostic and prognostic capabilities [51]. The development of explainable AI frameworks, multi-modal clinical data integration, and multi-task histological analysis capabilities represents the crucial pathway for translating computational pathology from research laboratories to routine clinical diagnostics.

*Pathological grading task* Weakly- and semi-supervised MIL frameworks have emerged as predominant approaches for pathological grading, significantly reducing dependency on fine-grained annotations while maintaining diagnostic accuracy across various cancer types. Recent advances include MIL frameworks utilizing ResNet-34 architecture for tumor grading with slide-level labels, demonstrating robust performance in grade differentiation and survival correlation [65]. Further innovation has led to graph convolutional network-based MIL (GCN-MIL), incorporating noise-filtering mechanisms to mitigate label variability [66]. The field has also seen semi-supervised algorithms like GasMIL that leverage MIL principles to enhance classification and grading accuracy [67]. Additionally, progressive frameworks have evolved from noise-rectifying architectures that address annotation inconsistencies to spatial-hierarchical models capable of multi-scale feature learning [188, 189]. These weakly-supervised approaches effectively address annotation scarcity while achieving diagnostic performance comparable to expert pathologists. Future advancement requires adaptive learning systems capable of handling variable annotation quality and establishing standardized evaluation protocols specifically for weakly-supervised pathological assessment to guarantee consistent clinical performance across diverse healthcare environments.

*LVI task* Current approaches for patch-based LVI detection primarily leverage weakly supervised learning paradigms to address the complex challenge of identifying tumor emboli within vascular structures. These methodologies focus on developing region-specific analytical frameworks that can effectively localize invasion patterns while operating under substantial annotation constraints. Recent developments include attention-based MIL frameworks that strategically leverage tumor tissue regions from WSIs. These systems identify diagnostically relevant areas through attention mechanisms and aggregate patch-level features to generate comprehensive slide-level predictions [61]. Building on this foundation, integrated dual-branch architectures have been designed to simultaneously handle classification objectives and cellular localization tasks. By combining patch-level feature extraction with spatial context modeling, these unified frameworks significantly enhance invasion pattern recognition capabilities [75]. These patch-level analytical methodologies demonstrate significant potential for precise vascular invasion assessment through localized feature learning while effectively minimizing annotation dependencies. Future advancement should prioritize the development of cross-cancer generalization frameworks capable of adapting learned representations from HCC to diverse cancer types exhibiting LVI patterns and multi-scale feature integration to expand the clinical applicability of these localized analysis approaches across various pathological contexts.

*LNM task* Current DL approaches for LNM prediction primarily employ attention-based MIL frameworks to analyze WSIs, enabling effective cancer staging through weakly-supervised whole-slide analysis. These methodologies demonstrate particular strength in extracting predictive features from primary tumor specimens across various cancer types. The technical implementation of these systems encompasses several sophisticated architectures. Specially designed attention-based MIL frameworks have been developed for core needle biopsy samples, utilizing deep feature representations to predict nodal status from limited tissue material [182]. Expanding beyond this approach, multiscale attention-based CNNs process H&E-stained pathological slides to preoperatively predict metastasis risk through integrated feature analysis across multiple magnification levels [85]. Further advancing clinical applicability, MIL-based AI systems now automatically assess LNM risk from WSIs, providing data-driven alternatives to traditional clinical algorithms for treatment planning [86]. These attention-based MIL methodologies collectively demonstrate the viability of weakly-supervised learning for accurate LNM prediction while significantly reducing dependency on detailed annotations.

*Quantitative feature-enhanced models* Recent advances in computational pathology have demonstrated the complementary value of integrating handcrafted quantitative features with DL architectures to enhance diagnostic precision. These hybrid approaches leverage both data-driven representations and domain-specific pathological knowledge to improve classification performance across various cancer types. One prominent methodology employs comprehensive feature engineering frameworks that systematically extract and combine multi-category descriptors, spanning local morphological characteristics, color properties, Haralick texture descriptors, and second-order Riesz features, which are subsequently processed through regularized classifiers for precise tumor differentiation [180]. An alternative architectural strategy implements dual-stage analytical systems that synergistically combine DL-based tumor assessment with traditional ML. In these systems, quantitative pathological metrics such as grade distribution percentages and cancer area measurements are processed through instance-based classifiers to generate final diagnostic determinations [68]. These hybrid methodologies effectively demonstrate that the strategic combination of engineered quantitative features and DL representations can surpass the performance of either approach individually, establishing a new paradigm for enhanced diagnostic accuracy in computational pathology.

Patch classification in pathology image analysis has established a robust foundation through the integration of handcrafted morphological features with traditional ML classifiers. For histological typing, studies have demonstrated that correlation-based feature selection combined with classifiers like decision trees, NB, and SVMs enables effective cancer subtype discrimination [51, 179, 186, 187]. In pathological grading, the development of visually interpretable luminal and structural features has proven particularly valuable, achieving high accuracy in prostate cancer grading through reconstruction error minimization and SVM-based classification [65–67, 188, 189]. These approaches consistently highlight the strength of combining biologically meaningful feature design with classical ML methods for precise diagnostic tasks at the patch level.

#### Detection-based pathology image analysis

Detection-based approaches in computational pathology employ fully supervised learning frameworks with specialized architectures to precisely localize and identify critical histological structures and cellular patterns. These methods primarily utilize region-based CNNs and encoder-decoder segmentation architectures to address 3 key diagnostic challenges: vascular invasion assessment, metastatic lesion identification, and immune cell quantification.

*LVI task* Current detection methodologies for LVI focus on precise vascular structure localization and characterization in standard H&E-stained images. Yi et al. [79] pioneered the application of fully CNNs for microvessel detection in HCC, establishing an automated framework that identifies clinically relevant vascular features correlated with patient outcomes. Their approach demonstrates the viability of DL-based vascular quantification for prognostic assessment in routine histopathological specimens.

*LNM task* LNM detection has evolved toward integrated cascaded architectures that combine region proposal networks with semantic segmentation models. Hu et al. [87] developed a multi-stage framework integrating Faster R-CNN for candidate region detection and DeepLab for precise boundary segmentation, complemented by feature extraction from Xception and DenseNet-121 models for metastatic classification. This comprehensive approach enables highly accurate quantification of lymph node involvement through sequential region localization and feature analysis.

*IHC task* IHC analysis utilizes detection models for automated immune cell quantification in stained tissue sections. Swiderska-Chadaj et al. [109] implemented DL algorithms trained on extensive datasets of manually annotated CD3^+^ and CD8^+^ lymphocytes to automatically detect and characterize immune cell populations in IHC images. Their methodology provides a robust foundation for the precise quantification of TME composition and immune response assessment.

Detection-based methods in computational pathology leverage fully supervised DL architectures to address critical diagnostic tasks through precise localization and identification of histological structures. These approaches primarily utilize region-based CNNs (e.g., faster regions with convolutional neural networks, Faster R-CNN) and encoder-decoder segmentation models (e.g., U-Net) to perform 3 essential functions: automated microvessel detection for LVI assessment [134], metastatic lesion identification in lymph nodes through cascaded detection-segmentation frameworks, and immune cell quantification in IHC images via specialized lymphocyte detection algorithms. The methodology demonstrates particular strength in combining multi-stage architectural designs with advanced feature extraction networks to achieve high-precision localization of diagnostically relevant regions and cellular patterns, significantly enhancing quantitative analysis capabilities in histopathological assessment.

#### Segmentation-based pathology image analysis

Whole-slide segmentation in computational pathology employs DL architectures to enable precise structural analysis across multiple biological scales [48]. Contemporary approaches leverage CNNs and encoder-decoder architectures to address fundamental diagnostic tasks, with U-Net variants demonstrating particular effectiveness in nuclear segmentation, glandular analysis, and biomarker quantification. These methodologies have evolved into integrated analytical frameworks that combine multi-modal data integration with automated annotation strategies to enhance diagnostic precision.

*Pathology image analysis task* Segmentation-based approaches in computational pathology have evolved to address the critical challenge of precise structural delineation through innovative multi-modal learning frameworks and automated annotation pipelines. These methodologies demonstrate particular effectiveness in leveraging complementary staining techniques to establish accurate reference standards while minimizing manual annotation burden.

Recent advances in nuclear analysis include distance prediction-based convolutional networks that achieve simultaneous nuclear segmentation and classification across diverse tissue types [69]. For glandular structure analysis, automated pipelines have been developed to transfer precise gland masks from IHC to corresponding H&E images [190]. This paradigm has been extended through two-step DL frameworks that first segment epithelial tissue in IHC-stained slides using specific biomarkers, then propagate these annotations to H&E images via image registration [191]. Beyond conventional staining methods, innovative pipelines employing immunofluorescence restaining with multiple antibodies have been introduced to generate unbiased segmentation labels for comprehensive cellular profiling [192]. Additional progress includes IHC-guided annotation systems that automatically detect inflammatory cells through dual staining and image registration [193], alongside automated label transfer pipelines that enable standardized tumor cellularity assessment between H&E and IHC modalities [194].

These segmentation frameworks collectively establish a new paradigm for histopathological analysis, demonstrating that multi-modal reference standards and automated annotation pipelines can effectively overcome traditional bottlenecks in digital pathology while maintaining diagnostic precision across diverse tissue types and disease conditions.

*IHC task* DL-based approaches have revolutionized immunohistochemical analysis by enabling precise biomarker quantification through advanced segmentation architectures and multimodal learning strategies. These methodologies address critical challenges in IHC evaluation, including stain variability quantification, cellular segmentation precision, and prognostic feature extraction.

Several innovative frameworks demonstrate this technological progress. For multiplex IHC analysis, U-Net based frameworks incorporating pathologists’ annotations have been implemented to quantitatively assess multiple biomarkers within tissue microenvironments, providing reliable cellular segmentation for spatial biomarker expression analysis [92]. To overcome technical variability in conventional IHC, multi-task learning architectures have been developed that leverage co-registered IHC and mIF data, effectively transforming standard IHC images into detailed mIF representations for enhanced cellular segmentation and protein quantification [97].

Advancing stain-specific quantification, automated algorithms utilizing hematoxylin staining characteristics enable accurate H-score evaluation, with validation across multiple cancer cohorts through comprehensive dataset development [100]. Expanding into prognostic applications, CNNs with autoencoder architectures extract high-dimensional pathological features from WSIs, establishing DL-based risk scoring systems for survival prediction validated through Kaplan-Meier analysis [104].

These DL frameworks collectively establish a new paradigm for quantitative IHC analysis, demonstrating that multimodal learning strategies and advanced segmentation architectures can effectively overcome traditional limitations in biomarker quantification while providing clinically relevant insights across multiple cancer types and staining protocols.

#### Regression-based pathology image analysis

Regression-based approaches in computational pathology have established powerful frameworks for predicting clinical outcomes through deep feature representation and sequential modeling architectures. These methodologies primarily leverage CNNs combined with specialized prediction heads to address 2 critical clinical domains: survival prognosis stratification and treatment response prediction across various cancer types.

In survival prognosis, several architectures have demonstrated significant predictive value. End-to-end frameworks integrating convolutional networks with survival analysis enable direct outcome prediction from histopathological images [112]. CNN-based systems have been implemented to automatically quantify proliferation activity through mitotic detection, providing features correlated with recurrence risk stratification [110]. Beyond cellular-level analysis, stromal quantification models identify TME composition features predictive of disease-free survival [123]. Scaling to larger datasets, ensemble CNN frameworks trained on millions of image tiles effectively stratify patients by clinical outcome [111]. Further extending this capability, deep neural network approaches have been developed to predict disease-specific survival across multiple institutional cohorts [120].

For treatment response prediction, diverse architectural strategies have emerged. Ensemble approaches combining EfficientNet, DenseNet, and swin transformer models generate immunotherapy response scores [183], while hybrid methodologies integrate VGG16 feature extraction with SVM classification to predict pathological complete response to neoadjuvant therapies [129]. In risk stratification, combining CNN-derived features with linear SVM enables patient classification into prognostic groups based on survival outcomes [121]. Advancing temporal modeling, recurrent neural network architectures sequentially process VGG16 feature vectors to predict long-term disease-specific survival from image sequences [122].

These regression frameworks collectively demonstrate that DL architectures can effectively extract clinically relevant prognostic information from histopathological images, establishing a new paradigm for quantitative outcome prediction in oncology that bridges computational analysis with clinical decision-making.

#### Quantitative feature extraction and utilization

Quantitative feature extraction methodologies in computational pathology bridge traditional feature engineering with DL by integrating domain-specific morphological characteristics with data-driven representations. These hybrid approaches leverage both handcrafted feature quantification and deep feature learning to enhance diagnostic precision, prognostic stratification, and treatment response prediction across multiple cancer types.

The methodological progression begins with feature engineering pipelines that extract cellular morphological and texture descriptors from WSIs. These handcrafted features are subsequently processed through ensemble ML classifiers to achieve accurate multi-class tissue region identification [73, 195]. Building upon this foundation, CNN frameworks further advance the quantification capacity by capturing global tissue architecture patterns and the spatial organization of cellular interactions. These DL-derived features, when combined with RF classification, enable the utilization of TME characteristics as robust diagnostic biomarkers [93].

Advancing to higher-order analytical capabilities, graph-based representations capture complex tissue spatial organization through structural topology features. This approach transforms histological patterns into quantifiable graph metrics that demonstrate predictive value for long-term survival outcomes [131]. Parallel developments in DL with contrastive clustering identify prognostic morphophenotypic clusters, generating integrated pathological signatures that simultaneously predict treatment response and patient survival [132].

Completing this methodological spectrum, recent architectures integrate nuclear segmentation with multi-category feature extraction. Weighted neural networks precisely segment cellular structures, then extract comprehensive feature sets encompassing morphological, texture, and graph-based descriptors. These multi-modal features subsequently train GBMs to predict therapeutic response, establishing an end-to-end analytical pipeline from cellular quantification to clinical outcome prediction [130].

These methodologies collectively establish that the strategic integration of quantitative feature engineering with DL architectures creates a synergistic framework where handcrafted morphological descriptors and data-driven representations complement each other to enhance both analytical performance and clinical interpretability across diverse pathological applications.

#### Other pathology image analysis tasks

Computational pathology has progressively advanced from basic diagnostic classification to sophisticated analysis of tumor biological characteristics. This evolution encompasses 3 interconnected analytical domains: necrosis quantification, TME characterization, and molecular correlation profiling, each employing distinct methodological frameworks to extract clinically relevant information from histopathological images.

Necrosis quantification methodologies have evolved through complementary technical strategies. Initial approaches employed interpretable multimodal frameworks that segment tumor cell nuclei and extract morphological features to analyze necrotic regions and their prognostic significance [71]. Subsequently, transfer learning strategies utilizing DenseNet architectures have been implemented to extract bottleneck features for automated necrosis detection, demonstrating the adaptability of deep features across tissue types [72].

TME characterization represents a more complex analytical domain, where multiple parallel developments have emerged. DL pipelines now enable automated immune infiltration quantification and TSR scoring, establishing correlations between stromal proportion and clinical outcomes [84]. Weakly supervised frameworks address annotation limitations by estimating stromal TIL percentages from slide-level labels [90], while spatial analysis methods extract and analyze spatial distribution patterns of immune cells, demonstrating prognostic relevance for survival outcomes [91]. For comprehensive profiling, integrated architectures combining color-aware autoencoders with U-Net networks analyze multiple cell types and stromal maturity through multiplex IHC [92].

Molecular correlation and subtyping constitute the most advanced application domain, bridging histology with molecular pathology. Deep residual learning approaches directly identify microsatellite instability from conventional histology slides, enabling molecular subtyping without additional testing [105]. Comprehensive diagnostic models further predict protein expression status and automatically classify tumors according to WHO standards, effectively bridging histopathological features with molecular characteristics [106].

These methodological advances collectively demonstrate a clear trajectory: from morphological quantification to spatial relationship analysis, and ultimately to molecular correlation. This progression has established computational pathology as an indispensable bridge connecting tissue morphology with biological behavior, significantly expanding the analytical dimensions beyond traditional diagnostic classification while enhancing reproducibility through quantitative assessment.

DL models, through the integration of architectural innovations and feature engineering, have established an analytical framework that covers the entire workflow of pathological diagnosis [48]. At the technical architecture level, CNNs and transformers are responsible for whole-slide classification, MIL handles patch-level analysis, region detection networks enable structural localization, encoder-decoder architectures accomplish fine-grained segmentation, and hybrid regression models facilitate prognostic prediction. The fusion of quantitative features and deep features is particularly innovative, leveraging both domain knowledge and data-driven approaches to demonstrate significant advantages in diagnostic accuracy, prognostic stratification, and treatment response prediction [155]. The field has evolved from basic classification to the in-depth extraction of biological information. Current technologies not only perform complex tasks such as TME analysis and molecular subtype inference but also establish quantitative correlations between pathological features and clinical outcomes through spatial distribution analysis and biomarker quantification. This paradigm shift has elevated computational models beyond auxiliary diagnostic tools, making them critical bridges connecting tissue morphology and biological behavior, thereby enhancing diagnostic reproducibility while significantly expanding the analytical dimensions of traditional pathology.

### Foundation model-based and multimodal models for pathology image analysis

In recent years, the emergence of large-scale pre-trained models has profoundly reshaped AI, driving notable progress in natural language processing, computer vision, image generation, and multimodal tasks. By leveraging massive datasets and self-supervised learning, researchers have developed highly transferable foundation language models [196, 197] and vision models [95, 198], while contrastive learning on paired image-text data has enabled multimodal models such as contrastive language-image pre-training (CLIP) [199] and sigmoid loss for language image pre-training (SigLIP) [200], with systems like chat generative pre-trained transformer (ChatGPT) further illustrating advanced cognitive abilities related to reasoning and memory [201, 202]. Despite these achievements, most general-purpose foundation models lack training and fine-tuning specific to pathology, limiting their direct utility in clinical and research applications. To bridge this gap, recent efforts have focused on developing pathology-oriented foundation models that adapt large-scale pre-training paradigms to domain-specific data, thereby improving the accuracy, efficiency, and robustness of pathology image analysis. Consequently, the application roadmap (Table 4) for these pathology-specialized foundation models is rapidly expanding to demonstrate their efficacy in critical tasks, ranging from slide-level classification and biomarker prediction to survival analysis and multimodal clinical integration [10, 51, 144, 203–214, 216–218]. In summary, while foundation models have already demonstrated transformative impact across diverse AI fields, their application in pathology remains an evolving frontier, where future work is expected to focus on building larger and more diverse pathology datasets, incorporating multimodal information such as text and molecular profiles, and improving interpretability and reliability to accelerate the clinical translation of foundation model technologies.

**Table 4:** Comparison of the roadmaps of large foundation models in digital pathology

Model type	Model name	Model size	Training data source	Training data size	Executable task	References
Self-supervised pre-training vision-only large foundation models	HIPT	28 M	TCGA	10,678 slides	Slide-level classification, survival prediction	[203]
	CTransPath	28 M	TCGA, PAIP	32,220 slides	Slide-level classification, patch-level classification, patch recall, mitosis detection, and colorectal adenocarcinoma gland segmentation	[204]
	BROW	86 M	Private data, TCGA	11,206 slides	Slide-level classification, patch-level classification, and nucleus segmentation	[205]
	UNI	303 M	Private data	100,426 slides	Slide-level classification, patch-level classification, cell type segmentation, and image recall	[144]
	Prov-GigaPath	1 B	Private data	171,189 slides	Slide-level classification, patch-level classification, and zero-shot classification	[10]
	Virchow	632 M	Memorial Sloan Kettering Cancer Center (MSKCC)	1.5M slides	Slide-level classification, rare cancer detection, biomarker prediction, and survival prediction	[206]
	Virchow2	632 M	MSKCC + diverse global institutions	3.1M slides	Slide-level classification, rare cancer detection, biomarker prediction, and survival prediction	[207]
	CHIEF	Unreleased	Public & private pathology data from 19 anatomical sites	60,530 slides, 15 M tiles	Slide-level classification, tumor origin prediction, genomic mutation prediction, and survival prediction	[51]
Visual language Contrast learning pre-trained large foundation models	MI-Zero	383 M	Open educational resource, ARCH	33 K image-text pairs	Pathology image classification, zero sample classification, and cross-modal recall	[209]
	CONCH	395 M	PubMed, Private data	1.17 M image-text pairs	Pathology image classification, zero sample classification, and cross-modal recall	[210]
	PLIP	151 M	Twitter, LAION	208 K image-text pairs	Pathology image classification, zero sample classification, and cross-modal recall	[211]
	QuiltNet	151 M	YouTube, PubMed, Twitter, LAION	1 M image-text pairs	Pathology image classification, zero sample classification, and cross-modal recall	[212]
	mSTAR	Unreleased	TCGA	26,169 pathological sections, Report, RNA-Seq data-pairs	Pathology image classification, zero sample classification, and survival analysis	[213]
	TITAN	48.5 M	Mass-340 K dataset (public and internal, 20 organs)	335,645 slides, 423,122 captions, 182,862 reports	Pathology image classification, biomarker prediction, survival prediction, report generation, and cross-modal retrieval	[208]
Multi-modal large foundation model based on large language models	PathAsst	13 B	PubMed, Internal Pathology teaching materials, Liquid-based cytology data specified by experts	207 K image-caption pairs, 180 K instruction-following data	Pathological visual question-and-answer, and auxiliary model invocation	[214]
	Quilt LLaVA	7 B	YouTube	723 K image-caption pairs, 107 K instruction-following data	Pathology visual question-and-answer, and pathology-assisted teaching	[216]
	PathChat	13 B	PubMed, Private data	100 K image−caption pairs, 457 K instruction following data	Pathological visual question-and-answer	[217]
	OmniPath	13 B	Public and private datasets, 20 organs	490,000 samples	Cancer detection, grading, vascular/neural invasion, prognosis, segmentation, referring expression, and visual question-and-answer	[218]

*TCGA* The Cancer Genome Atlas, *HIPT* Hierarchical image pyramid transformer, *PAIP* pathology artificial intelligence platform*, BROW* better features for whole slide image*, MI-Zero* multiple instance zero-shot transfer for histopathology images*, ARCH* autoregressive conditional heteroskedasticity model, *MSKCC* Memorial Sloan Kettering Cancer Center, *LAION* large-scale artificial intelligence open network, *CHIEF* clinical histopathology imaging evaluation foundation, *CONCH* contrastive learning from captions for histopathology, *TITAN* transformer-based pathology image and text alignment network, *PLIP* pathology language-image pre-training, *mSTAR* multimodal self-taught pre-training

#### Self-supervised pre-training vision-only large foundation models

Some studies have collected large public or private pathology image datasets and used self-supervised methods to train their own foundation models for pathology images [203–206]. As image encoders, these models are utilized to extract features from pathology images and adapt them to downstream pathology tasks through transfer learning and other methods, achieving results that surpass traditional pathology image models.

Recently, several pathology-specific foundation models have been proposed to further enhance pathology image analysis by improving model accuracy, expanding the range of downstream tasks, and increasing robustness across heterogeneous data sources. Virchow [206] is a 632-million-parameter vision transformer trained on approximately 1.5 million H&E-stained WSIs from 119,629 patients treated at Memorial Sloan Kettering Cancer Center. It uses the DINOv2 self-supervised learning framework to generate transferable embeddings for diverse pathology tasks, including slide-level classification, rare cancer detection, biomarker prediction, and survival analysis, achieving an area under the curve (AUC) of 0.95 for pan-cancer detection. Virchow2 [207] expands the dataset to over 3.1 million WSIs from 225,401 patients across globally diverse institutions, incorporates mixed magnifications and multiple staining types, and applies pathology-specific algorithmic improvements. It shows superior performance on both in-domain and out-of-domain benchmarks across tile- and slide-level tasks. The UNI model [103] is a self-supervised learning model trained on a large-scale pathology dataset, demonstrating outstanding performance across 34 computational pathology tasks, particularly in histopathological classification and disease subtype prediction. The Prov-GigaPath [10] was trained on over 1.3 billion pathology image patches from more than 170,000 WSIs, highlighting the potential of large-scale pathology model training. Another notable model, the clinical histopathology imaging evaluation foundation (CHIEF) model [51], has been designed to detect multiple cancer types with high accuracy, evaluate treatment efficacy, and predict patient survival, achieving a diagnostic accuracy of up to 96% in certain cancer types.

#### Vision-language contrastive pre-trained large foundation models

In addition to vision-only encoders, contrastive learning frameworks have been widely adopted to pre-train large vision-language models in pathology. By aligning histopathology images with their associated reports or captions, these models enable zero-shot classification, image-text retrieval, and survival prediction tasks.

A representative example is the transformer-based pathology image and text alignment network (TITAN) [208], which aligns histopathology images with their pathology reports, supporting zero-shot cancer retrieval and survival prognosis prediction. Similarly, visual language pretrained multiple instance zero-shot transfer for histopathology images (MI-Zero) [209] applies contrastive pre-training to enable zero-shot multiple-instance learning, allowing classification and retrieval without task-specific fine-tuning and demonstrating adaptability in rare disease scenarios. The contrastive learning from captions for histopathology (CONCH) [210] model extends this approach by utilizing large-scale paired pathology image-text data, improving WSI-level classification and retrieval while ensuring scalability across institutions. Pathology language-image pre-training (PLIP) adapts the CLIP framework specifically for pathology datasets, showing superior performance in zero-shot classification and retrieval tasks, thereby highlighting the importance of domain-specific contrastive pre-training [211]. QuiltNet further advances this line of research by integrating diverse image-caption datasets, enabling robust cross-domain generalization in heterogeneous pathology data [212]. Meanwhile, multimodal self-taught pre-training (mSTAR) emphasizes multi-scale alignment between pathology images and textual descriptions, enhancing fine-grained retrieval performance for cancer subtype recognition and lesion-level annotation [213]. Collectively, these vision-language contrastive pre-trained foundation models demonstrate the feasibility of using paired image-text data to capture semantic information in pathology.

#### Multi-modal large foundation models based on large language models

With the rapid advancement of multimodal learning, an increasing number of studies have explored integrating pathology images with textual and molecular data to enhance AI-driven pathology applications. Expanding upon the use of multimodal data, the transcriptomics-guided slide representation learning model has been introduced to integrate transcriptomic (next-generation RNA sequencing) data with WSIs, improving slide-level representation learning [216]. Similarly, PathChat, a vision-language copilot for pathology, incorporates a foundation pathology image encoder with a large language model, allowing interactive AI-driven assistance for both diagnostic and educational applications in pathology [217]. These innovations demonstrate the potential of combining histopathology images with text and molecular data to improve AI-driven pathology models.

OmniPath is a large vision-language model for pathology, designed to enhance multiscale feature extraction in WSIs [218]. By integrating task-guided feature enhancement and prompt-guided feature completion, it achieves superior accuracy in cancer detection, grading, and pathology visual question answering, offering a more efficient AI-assisted diagnostic tool.

In addition to the purely image-based field, some studies have gathered a large amount of paired pathology image-text data from sources such as public educational resources and the PubMed database [209, 210], Twitter [211], and YouTube [212]. However, these foundation models often lack training and fine-tuning specific to the pathology domain, making them challenging to apply directly to pathology-related tasks. Therefore, many studies have focused on applying these foundation models in pathology. After data cleaning and processing, they trained their own multimodal models using a CLIP-like image, text contrastive learning approach [213–218]. These models, which incorporate textual information, not only accomplish the aforementioned pathology tasks through transfer learning but also exhibit better zero-shot capabilities compared to purely image-based models, enabling them to classify pathology images without requiring training samples. Furthermore, these models possess cross-modal retrieval and recall abilities, allowing them to extract images related to specific pathology concepts.

Additionally, foundation models like RudolfV et al. [219] have been developed specifically with pathology domain knowledge in mind, incorporating expert-driven curation strategies to improve model robustness across different staining protocols and cancer types. Going a step further, the mSTAR model [213] compiled a tri-modal dataset from The Cancer Genome Atlas (TCGA) that includes pathology slides, pathology reports, and next-generation RNA sequencing data. By utilizing this dataset for multimodal contrastive learning, the resulting model significantly enhances performance in tasks that require both genetic and textual information, such as molecular prediction and survival analysis. The success of ChatGPT has spurred the rapid development of generative large language models, laying the foundation for multimodal large models such as the large language and vision assistant (LLaVA) [220]. Inspired by the LLaVA approach, studies [54, 214, 215] have utilized cleaned paired pathology image-text data to generate visual instruction fine-tuning data with the aid of ChatGPT, and then fine-tuned the LLaVA model using this data. These models can perform visual question-and-answer tasks specific to pathology images, assist physicians in diagnosis, and can be applied in pathology education.

Recent surveys on pathology foundation models have also highlighted the growing field of computational pathology foundation models [221–223], reviewing state-of-the-art datasets, adaptation strategies, and evaluation benchmarks. These studies highlight key challenges, such as dataset standardization, model transparency, and the need for domain-specific fine-tuning, to enhance clinical applicability [221]. Overall, the rapid progress of multimodal pathology foundation models demonstrates their potential to revolutionize diagnostic workflows, education, and precision medicine, yet future research must address standardization, interpretability, and clinical validation to enable broader real-world adoption [224].

**Fig. 4:**
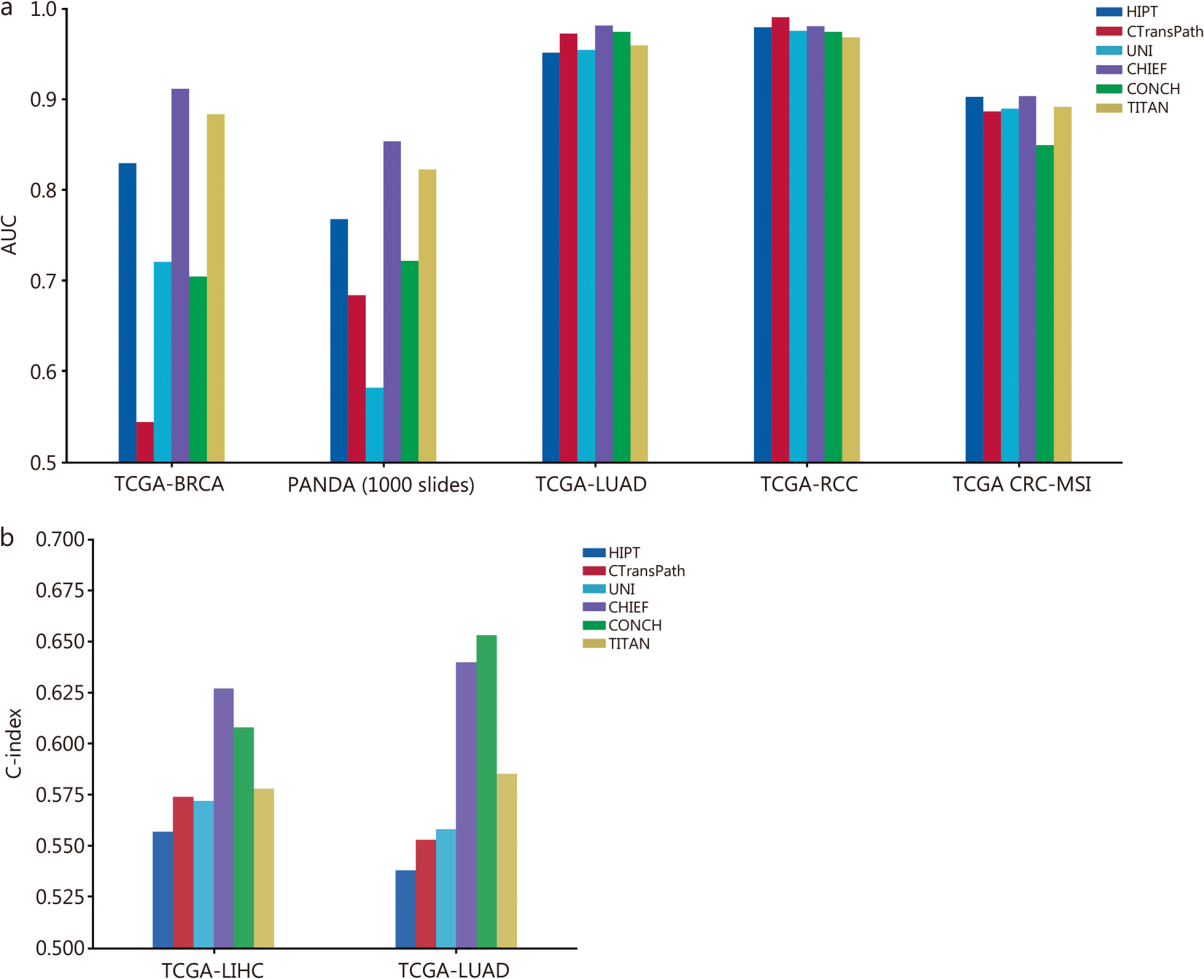
Performance comparison of pathological large models across diagnosis classification tasks. **a** The bar chart illustrates the area under curves (AUCs) across multiple cancer datasets. **b** The bar chart illustrates the C-index on TCGA-LIHC and TCGA-LUAD datasets. Each model is represented by a different color. The results highlight the diagnostic capabilities of each model across diverse pathological contexts. HIPT hierarchical image pyramid transformer, UNI towards a general-purpose foundation model for computational pathology, CHIEF clinical histopathology imaging evaluation foundation, CONCH contrastive learning from captions for histopathology, TITAN transformer-based pathology image and text alignment network, TCGA The Cancer Genome Atlas, BRCA breast cancer, LUDA lung adenocarcinoma, RCC renal cell carcinoma, CRC colorectal cancer, MSI microsatellite instability, LIHC liver hepatocellular carcinoma

To evaluate the comparative advantages of different foundation models, we conducted a comprehensive benchmark study assessing pathological large models on diagnosis and prognostic tasks across multiple cancer datasets (Fig. 4; Additional file 1: Tables S1, S2). The comparative analysis reveals that the visual foundation model CHIEF [51] achieves the best overall performance. The fundamental advantage of this model stems from its gating mechanism, which effectively filters out numerous irrelevant tissue patches, thereby enhancing the learning of task-relevant pathological features. The evolution of large pre-trained models has catalyzed the development of pathology-specific variants that are fine-tuned and optimized for diverse pathology applications. Multiple studies have utilized self-supervised learning on extensive pathology datasets to create models exhibiting robust generalizability and diagnostic capability across both classification and prognostic tasks [128, 224–227]. The experimental results presented here validate the efficacy of pathological large models across varying cancer types and task categories, providing valuable insights for advancing AI-driven pathology.

### The role of AI/ML in precision medicine

Within the framework of malignant tumor diagnosis and treatment systems, AI and ML technologies are increasingly becoming vital tools for overcoming the limitations of traditional pathological diagnosis methods. They significantly enhance precision, efficiency, and objectivity in cancer screening and diagnosis, prognostic prediction, and biomarker discovery.

In digital pathology, the use of AI and ML models to analyze high-resolution WSIs has markedly improved the accuracy of tumor screening and diagnosis. Traditional pathological diagnosis relies on physicians’ expertise and is prone to subjective variability. In contrast, DL algorithms (e.g., CNNs) can automatically identify histological features such as cellular atypia, mitotic figures, and tumor infiltration patterns [99]. Additionally, AI-powered real-time pathological analysis shortens diagnostic turnaround times, particularly benefiting resource-limited regions by facilitating early tumor detection and medical intervention [101]. For example, integrating ML with intelligent analysis of multi-omics data from liquid biopsy samples enabled non-invasive early detection of ovarian cancer and risk stratification for high-risk populations, offering physicians diagnostic tools with higher sensitivity and specificity [224]

Combining digital pathology with AI transcends conventional histopathological grading by quantifying TME features to predict patient outcomes. Temporal analysis of pathology images also enables dynamic assessment of treatment response. For example, AI-based quantification of tumor regression patterns after neoadjuvant chemotherapy (NAC) provides objective evidence for clinical decision-making, supporting personalized treatment optimization. For example, Amgad et al. [228] constructed the histomic prognostic signature (HiPS) scoring system, employing DL models to automatically extract key features from TME components, such as epithelial, immune, and stromal cells, in histopathological slides. The HiPS scoring system has significantly improved the ability to predict the prognosis of patients with breast cancer, surpassing the performance of traditional subjective scoring systems. Traditional biomarker discovery relies on manual feature extraction, which restricts its overall effectiveness. In contrast, AI employs unsupervised learning (e.g., clustering and autoencoders) to mine associations between morphological features and molecular aberrations from vast WSIs. This enhances the effectiveness of AI in diagnosis, while these discoveries not only advance the development of bimodal morphological-molecular biomarkers but also the identification of new therapeutic targets, accelerating the translation of precision medicine into clinical practice. In one such application, Luo et al. [106] developed a DL model to predict prognosis and then combined this information with a deep representation clustering technique to discover phenotypic markers in pathology images.

## Open datasets for pathological analysis

Open datasets are essential for advancing pathology image analysis. To this end, a curated summary of open datasets [10, 68, 69, 78, 80, 83, 109, 162, 193, 194, 212, 229–242] for pathology image analysis is presented (Table 5). It compiles several open datasets in the field of pathology image analysis, covering pathology image data from different organs, primarily using H&E staining. These datasets include image data at various magnifications, with sizes ranging from hundreds to hundreds of thousands of images. The data types include WSIs and image patches, providing annotations for various tasks, such as classification, segmentation, and prognosis, assisting researchers in selecting appropriate datasets for different pathological research and applications. Overall, this provides a concise overview of widely used open datasets in pathology image analysis.

**Table 5:** Summary of existing open datasets for pathology image analysis

Dataset name	Organs	Staining	Magnification	Size	Type	Data	Task	References
CAMELYON16	Lymph node	H&E	–	400 patients	WSI	Mask	Classification, segmentation	[80]
CAMELYON17	Lymph node	H&E	–	1000 patients	WSI	Mask	Classification, segmentation	[229]
Lymphocyte Detection	Lymph node	H&E	–	199 patients	WSI	Mask	Classification, segmentation	[230]
TCGA	Prostate	H&E	40 ×	397 patients	WSI	Label	Prognosis	[68]
TCGA	Prostate	H&E	20 ×	55 patients	WSI, Patch	ROI	Classification	[162]
TCGA	Lymph node	H&E	2.5 × , 5 × *,* 20 × *,* 40 ×	386 patients	WSI, Patch	Label	Classification	[83]
LYON19	Breast, colon, prostate	H&E	–	171,166 cells	Patch	ROI	Cell detection	[109]
BreaKHis	Breast	H&E	40 × , 100 × *,* 200 × , 400 ×	7909 images	Patch	Label	Classification	[231]
BACH	Breast	H&E	–	400 patients	WSI, Patch	Label	Classification, segmentation	[232]
LC25000	Lung, colon	H&E	60 ×	25,000 images	Patch	Label	Classification	[233]
TCGA	Microvessel	H&E	5 × , 10 × , 20 × , 40 ×	488 patients	WSI, patch	Label	Classification	[78]
Quilt-1M	Multiple	H&E	–	768,826 image-caption pairs	Patch	Label	Classification	[211]
PCam	Lymph node	H&E	–	327,680 images	Patch	Label	Classification	[10]
PANDA	Prostate	H&E	–	10,616 images	WSI, patch	Label	Classification	[234]
ARCH	Multiple	Multiple	–	4270 image-caption pairs	Patch	Label	Representation learning	[235]
PAIP	Liver	H&E	20 ×	100 patients	WSI	Mask	Segmentation	[236]
MoNuSeg	Multiple	H&E	40 ×	44 images (30 train + 14 test)	Patch	Mask	Segmentation	[237]
MoNuSAC	Lung, prostate, kidney, breast	H&E	40 ×	71 patients (46,000 nuclei)	Patch	Mask	Segmentation, classification	[238]
CoNSeP	Colon	H&E	40 ×	24,319 nuclei	Patch	Mask	Segmentation, classification	[69]
NeuLy‑IHC	Colon	H&E / IHC	40 × /40 ×	519 ROIs (235,256 cells)	Patch	ROI	Segmentation, classification	[193]
NuInsSeg	31 human & mouse organs	H&E	–	665 patches (30,698 nuclei)	Patch	Mask	Segmentation	[194]
TNBC	Breast	H&E	40 ×	50 images (4,022 nuclei)	Patch	Mask	Segmentation	[239]
TUPAC16	Breast	H&E	20 ×	821 images	WSI	ROI	Classification	[240]
GlaS	Colon	H&E	20 ×	165 images	Patch	Mask	Segmentation	[24]
MITOS‑ATYPIA‑14	Breast	H&E	40 ×	4240 images	Patch	ROI	Detection	[242]

“-” indicates that magnification information is not provided. *TCGA* The Cancer Genome Atlas, *LYON19* Lymphocyte Detection, *BreaKHis* breast cancer histopathological database, *BACH* breast cancer histology images, *LC25000* lung and colon cancer histopathological images, *PCam* PatchCamelyon, *ARCH* autoregressive conditional heteroskedasticity model, *MoNuSeg* multi-organ nucleus segmentation, *MoNuSAC* multi-organ nuclei segmentation and classification challenge, *NeuLy-IHC* neutrophil lymphocyte and immunohistochemistry dataset, *NuInsSeg* nuclei instance segmentation, *TNBC* triple-negative breast cancer, *TUPAC16* tumor proliferation assessment grand challenge, *GlaS* gland segmentation, *WSI* whole-slide image, *TCGA* The Cancer Genome Atlas, *H&E* hematoxylin and eosin, *PAIP* pathology artificial intelligence platform, *ROI* region of interest

## Challenges in intelligent pathological analysis

### Giga-pixel pathology images result in significant training and inference time costs

Pathology images typically contain up to tens of billions of pixels, and their extremely large size results in very long processing times for both model training and inference. Existing methods for pathology images typically use cropped image patches for feature extraction and model prediction, resulting in inference times of hundreds of seconds per slide [243, 244]. Inefficient pathology image analysis severely limits the clinical application of these methods. Designing faster model inference strategies for pathology image features is a key prerequisite for truly applying intelligent pathology diagnostic models to clinical diagnostic assistance in the future [186].

### Difficulty in precise annotation and the presence of substantial label noise

A critical challenge in intelligent pathology diagnostics is the reliance on large amounts of high-quality annotated data to train and validate models. However, pathology image annotation is highly demanding, and the scarcity of accurate labels significantly limits model generalizability and clinical applicability. The difficulty arises from several factors. First, annotating pathology images is time-consuming and requires detailed input from expert pathologists, while publicly available datasets remain limited in scale and quality [154, 245, 246]. Due to the extremely large size of WSIs and the subjectivity of cancer grading and subtyping, most datasets contain imprecise or incomplete annotations [188]. Second, variations in staining protocols and imaging conditions across laboratories alter the appearance of pathological features, introducing additional label noise [28]. Third, demographic imbalances in datasets may result in biased models that perform well for certain populations but underperform for others, exacerbating healthcare disparities. Furthermore, inconsistent annotations between pathologists increase subjectivity and further amplify the challenge of reliable ground truth.

Addressing these issues requires both methodological and data-centric solutions. Existing approaches to resist label noise primarily focus on designing loss functions or sample selection strategies to reduce the impact of incorrect labels, yet these methods often treat all noise as homogeneous and cannot fully resolve the problem. More effective strategies should aim at the root causes of pathology noise, incorporating noise-resistant model designs and robust training paradigms. At the data level, alternative sources of supervision can mitigate annotation scarcity. For instance, IHC-stained slides provide high-quality, objective labels without requiring manual annotation [42, 92], while clinical outcomes offer broader perspectives for model training, though they may introduce confounding factors. Combining pathologists’ specific annotations with objective data such as IHC labels [206] and outcome information, while balancing their respective strengths and limitations, is crucial for improving the reliability, robustness, and clinical relevance of AI models in pathology.

### Challenges in associating multi-level features and various factors in pathology image analysis

In clinical cancer diagnosis tasks, the assessment of patients’ prognoses typically requires a careful consideration of various factors, such as tumor grading, cancer cell spread, and chronic diseases, which are highly correlated with multiscale features in pathology images, including slices, tissues, and cells [247, 248]. However, existing pathology image diagnostic methods only predict from a single scale or concatenate multiscale features, lacking interlevel associations, which leads to the omission of important features related to cancer, thus limiting the performance of the final cancer prognosis prediction model. Effectively extracting multiscale features related to cancer prognosis and realizing the association and complementarity between them to provide comprehensive evaluation information for cancer prognosis prediction remains a challenge in intelligent pathology diagnostics [141, 186].

### Poor model interpretability and the presence of clinical ethical concerns

Although DL models perform excellently in pathology diagnostics, their black-box nature poses a significant issue in their interpretability. In clinical auxiliary diagnostics, physicians not only know the diagnostic results but also understand the reasons behind the model’s predictions [45, 75, 144, 226]. Therefore, improving model interpretability and providing transparent diagnostic bases are crucial for enhancing physicians’ trust and practical application value. Additionally, the poor interpretability of deep models raises ethical concerns in clinical applications, including data privacy protection and the attribution of diagnostic responsibility [249]. These concerns directly relate to patients’ vital interests in the promotion and application of technology. Therefore, promoting the development of intelligent pathology diagnosis technology while considering and addressing related ethical and legal concerns remains another major challenge faced by intelligent pathology diagnostics.

### Lack of quantification of model uncertainty and challenges in domain adaptation

For intelligent pathology diagnostic systems to be clinically adopted, the confidence level of their AI-powered predictions should be understood. Techniques such as Bayesian DL and ensemble methods can quantify model uncertainty, offering a measure of confidence that can enhance the interpretability and reliability of AI-assisted diagnoses.

Additionally, variability in pathology data across different institutions, scanners, and staining procedures introduces domain shifts that can degrade model performance when applied to new datasets [31, 37–40]. Models trained on data from a specific source may not generalize well to data from other sources due to these discrepancies. Domain adaptation techniques, including transfer learning and self-supervised learning, can improve model generalization by adapting models to new domains without requiring extensive labeled data [221, 244]. By addressing both uncertainty quantification and domain adaptation, intelligent pathology image analysis can become more robust and widely applicable across various clinical settings.

## Future direction for intelligent pathology image analysis

As previously discussed, while intelligent pathology image analysis technologies have great potential in improving diagnostic efficiency and accuracy [186, 189], they also face significant challenges. Future research and applications must focus on addressing issues such as the time-consuming nature of large pathology images [186], insufficiently precise data annotation [188], multiscale feature integration [141], model interpretability [45, 75, 144, 226], and ethical and legal concerns [249]. Only by overcoming these challenges can intelligent pathology image analysis technologies be widely applied in clinical practice, ultimately achieving more accurate cancer diagnosis assistance. Based on the current state of research summarized above, the future directions for intelligent pathology image analysis may include the following:

### Efficient training and inference framework for WSI analysis

Given the issue of long diagnostic times associated with current intelligent pathology image analysis technologies, achieving faster cancer diagnoses is a vital development direction. Key research areas include data compression of WSIs, accurate localization of diagnostically relevant regions, and acceleration of model training and inference. At the data level, it is necessary to compress WSIs while retaining microscopic scale features to reduce storage needs and improve the efficiency of subsequent model training and diagnosis prediction. In terms of region selection, focusing on key areas can effectively exclude irrelevant or redundant parts, achieving more efficient diagnostic predictions. At the model level, extensive research on CNNs and self-attention mechanisms can be combined with the characteristics of pathological data to achieve more efficient training and inference. These directions can all lead to faster feature extraction and cancer prediction, further promoting more efficient intelligent pathology image analysis. Enhancing model efficiency is a key prerequisite for applying this technology in clinical diagnostics, making the rapid diagnosis of cancer from WSIs a significant goal for developing future intelligent pathology image analysis technologies.

In addition to algorithmic efficiency, data acquisition strategies also influence diagnostic performance. Traditional WSI usually captures a single focal plane, which may miss fine-scale 3D or overlapping structures. Multilayer z-stack scanning records several focal depths, providing richer axial context for both pathologists and AI models. Existing study has shown that z-stacks improve the recognition of features such as mitotic figures and overlapping nuclei, enhancing classification and detection performance [250]. However, this technique also increases scanning time, storage,

and computational demands, which remain barriers to large-scale use. Future research should focus on task-specific depth selection, efficient compression of multilayer data, and model architectures that leverage axial cues, thereby achieving diagnostic gains while maintaining clinical feasibility.

### Noise-resistant framework for pathology image analysis

Existing noise-resistant methods are primarily applied to natural images, with few tailored to the characteristics of pathology images. Some noise-resistant models have been designed based on the causes of noise labels in pathology images; however, existing noise-resistant model designs still have limitations. The future development of intelligent pathology image analysis technologies will involve in-depth research on the characteristics of noise labels in pathology images, as well as designing targeted noise-resistant frameworks based on the fundamental causes of noise labels in pathological images. For example, such developments should address errors in cancer region annotation caused by difficulties in precisely segmenting cancers at the spatial level, and reduce grading annotation bias caused by subjective differences among physicians at the level layer. Combining these characteristics can lead to more robust intelligent pathology image analysis strategies.

Additionally, digital pathology datasets often vary significantly in terms of image quality, staining protocols, and labeling criteria across institutions, which introduces significant challenges. Future research should focus on addressing these inter-institutional differences. For example, models could be developed that standardize staining variations, such as differences in color intensity caused by varying staining protocols, and adjust for image quality discrepancies, including resolution and focus, thereby effectively mitigating the impact of dataset heterogeneity.

Since label noise is an almost unavoidable problem in pathology image data annotation, designing noise-resistant frameworks tailored to the characteristics of pathology images will be a vital direction for achieving intelligent cancer grading. In-depth research and resolution of the fundamental issue of label noise in pathology images can lead to more accurate and robust intelligent pathology image analysis technologies, thereby improving the precision and reliability of their cancer diagnoses.

### Large foundation models for multiscale pathology image analysis

Clinical pathology image analysis typically requires evaluating multiple indicators, including multiscale information from the slice scale to tissue and cell scales. However, most existing research on cancer grading from pathology images achieved predictions at a single scale or via multiscale concatenation, which is limited in performance and fails to meet the needs of assisting physicians in cancer diagnosis in clinical practice. Due to the limited annotations in most existing pathology image datasets, existing models struggle to accurately extract multiscale features for different diagnostic training tasks. Future research should focus on the comprehensive evaluation and diagnosis of cancer at different scales in pathology images based on richer pathological datasets with more annotations. Additionally, to enhance the practical application and reliability of models in clinical settings, multiscale features (e.g., slices, tissues, and cells) should be integrated with clinical diagnostic information to construct large foundation models for diagnosing cancer from pathology images.

In summary, obtaining richer annotated data and integrating multiscale features and clinical information are expected to achieve intelligent cancer diagnosis frameworks that can be truly applied in clinical practice. Such frameworks will significantly enhance the accuracy and efficiency of pathology image analysis, providing physicians with powerful auxiliary tools and ultimately improving the quality of cancer diagnosis and treatment.

### Interpretable models for intelligent pathology image analysis

The interpretability of current intelligent diagnostic models for pathology images remains in its infancy. Despite some progress being made in diagnostic performance, most existing models have significant deficiencies in result interpretation and credibility. Existing research on the interpretability of deep models has primarily focused on natural image data, lacking interpretable model designs tailored to the characteristics of pathological data. Since physicians and patients must understand and trust the model’s diagnostic results, designing interpretable pathological models is crucial for diagnosis. In the future, the design of interpretable models for intelligent pathology image analysis will develop towards more in-depth and precise directions, including models that can provide detailed diagnostic processes and bases, enabling physicians and patients to intuitively understand how they reach diagnostic conclusions. For example, they could integrate visualization tools to display the region of interests (ROIs) and features extracted by the model in pathology images, and use interpretability algorithms to reveal the logic and data-driven factors behind their decisions. Additionally, future research could combine clinical, genetic, and other multimodal data to provide more comprehensive and enriched diagnostic bases, thereby enhancing diagnostic accuracy and reliability.

In summary, enhancing the interpretability of intelligent diagnostic models for pathology images represents a crucial development direction. Continuous improvement and optimization of the interpretability of model designs will ensure that intelligent diagnostic systems are widely accepted and applied in clinical practice, ultimately providing patients with higher-quality medical care.

### Development of lightweight models and utilization of edge computing, cloud computing, and federated learning for resource-limited environments

As intelligent pathology image analysis technologies advance, their application in diverse clinical settings, including resource-limited settings, becomes increasingly important. Traditional DL models often require substantial computational resources and high-performance hardware, which may not be readily available in all medical facilities. Developing lightweight models optimized for efficiency and capable of running on less powerful systems will enhance the feasibility of deploying AI-powered solutions in these settings. Edge computing enables data processing to occur closer to the data source, reducing latency and reliance on continuous internet connectivity, which is particularly beneficial in remote or under-resourced settings. Cloud computing offers scalable resources for storage and computation, enabling institutions to leverage powerful analytics without significant investments in local infrastructure. Federated learning provides a framework for training models across multiple institutions without sharing sensitive patient data, addressing privacy concerns and legal restrictions while improving model robustness through diverse data exposure. By integrating these technologies, future research can focus on creating AI models that are not only effective but also accessible and practical for diverse medical settings, thereby enhancing the overall impact of intelligent pathology image analysis in global healthcare.

### Comparative evaluation of supervision strategies and automated parameter optimization frameworks

Fully supervised, semi-supervised, and self-supervised methods each have their own advantages and limitations in pathology image analysis. Fully supervised methods achieve high accuracy with large annotated datasets but require intensive manual labeling. Semi-supervised methods reduce annotation costs by utilizing unlabeled data, while self-supervised methods can be pre-trained on large unlabeled datasets and show strong generalization. In addition, current frameworks lack automated optimization of critical parameters, such as input size, magnification, and network complexity, which are often manually set, reducing efficiency and limiting model adaptability. Future research could focus on developing intelligent frameworks that automatically adjust these parameters using adaptive algorithms and optimization techniques, thereby improving robustness and making intelligent pathology image analysis systems more applicable in clinical practice.

### Multidimensional extension of generative models in pathology image analysis

Generative models are gaining increasing attention in digital pathology due to their ability to learn the joint distribution of complex visual and semantic patterns, thereby enabling realistic image synthesis, cross-modality translation, and context-aware interpretation under weak or even unsupervised supervision. Compared to traditional discriminative approaches that focus primarily on prediction tasks, generative models offer richer modeling flexibility by reconstructing underlying data distributions, which proves particularly valuable in scenarios with heterogeneous staining protocols, limited annotations, or domain shifts.

At the visual level, various generative paradigms, such as GANs, variational autoencoders, and diffusion models, can be employed to achieve virtual staining transformations, including modality translations, such as H&E to IHC, mIF, or other clinically relevant staining protocols, thereby facilitating multimodality learning, data augmentation, and stain normalization. At the semantic level, integrating generative models with large-scale vision-language architectures enables the automated generation of diagnostic reports, lesion-level textual explanations, and interactive clinical question-and-answer. Future directions may involve combining generative models with structural priors, domain-specific knowledge graphs, and contextual awareness in real-world clinical workflows.

### Multi-omics facilitates the precision diagnosis and treatment of tumors

Tumor development and progression involve intricate molecular mechanisms and TME heterogeneity, which cannot be fully deciphered using unimodal data. Multi-omics data, encompassing radiomics, genomics, proteomics, metabolomics, and liquid biopsy, collectively capture tumor characteristics across macro-morphological, molecular regulatory, and functional metabolic dimensions. By leveraging AI-powered multimodal integration, these high-dimensional heterogeneous datasets can be synthesized to construct cross-scale tumor feature maps, enabling more accurate disease subtyping, prognosis predictions, and treatment response assessments. Correlating pathological phenotypes with molecular signatures also facilitates the systematic discovery of critical biomarkers, revealing tumorigenic mechanisms and guiding the selection of personalized therapeutic targets. With advancements in multi-omics data integration algorithms and interpretable modeling, intelligent pathology will drive oncology toward multidimensional, dynamic, and precision-based diagnostics and therapeutics, ultimately transitioning from decision support to comprehensive, AI-enhanced disease management.

### Ethical and privacy safeguards for AI in pathology

As intelligent pathology imaging advances toward clinical implementation, addressing patient privacy and ethical governance becomes crucial. The primary challenge lies in preventing patient re-identification, as WSIs contain unique morphological patterns that can serve as biometric fingerprints, potentially enabling identity linkage across datasets through AI models.

A dual approach is essential to mitigate these risks. Technically, strategies such as tile-based data release, federated learning, and differential privacy can enhance protection. Administratively, implementing graded data access, standardized risk assessments, and clear data usage agreements are equally important. Furthermore, establishing robust ethical and legal frameworks is imperative. This includes ensuring compliance with regulations, obtaining informed consent, and collaborating with regulatory bodies to develop clear approval pathways for AI tools.

By integrating these technical and governance safeguards, the field can effectively balance AI innovation with patient rights, fostering trust and facilitating the responsible clinical integration of intelligent pathology systems.

## Conclusions

This review systematically traces the evolution of intelligent pathology image analysis, showcasing how AI, from traditional ML to DL and foundation models, has revolutionized diagnostic workflows. By integrating quantitative features with data-driven architectures, these technologies enable precise tumor classification, microenvironment characterization, and biomarker discovery while enhancing interpretability and reproducibility. Despite persistent challenges in scalability, annotation quality, and clinical translation, the convergence of multimodal data integration, noise-resistant frameworks, and ethically aligned design promises to bridge computational advances with real-world practice. Ultimately, intelligent pathology systems are poised to redefine precision oncology by delivering standardized, accessible, and actionable insights from histopathological data.

## Supplementary Information

**Additional file1.** **Table S1** Comparison of pathological large models on classification tasks across multiple cancer datasets.** Table S2** Comparison of pathological large models on prognostic tasks across cancer datasets.

## Data Availability

Not applicable.

## Abbreviations

AI: Artificial intelligence
ANN: Artificial neural network
ARCH: Autoregressive conditional heteroskedasticity model
AUC: Area under the curve
BRCA: Breast cancer
BACH: Breast cancer histology images
BROW: Better features for whole slide image
BreaKHis: Breast cancer histopathological database
CNN: Convolutional neural network
ChatGPT: Chat generative pre-trained transformer
CGA: Cellular and glandular architectural
CoNSeP: Colorectal nuclear segmentation and phenotypes dataset
CRC: Colorectal cancer
CHIEF: Clinical histopathology imaging evaluation foundation
CLIP: Contrastive language-image pre-training
CONCH: Contrastive learning from captions for histopathology
ConViT: Convolutional-like vision transformer
CRC: Colorectal cancer
DL: Deep learning
DNN: Deep neural network
ER: Estrogen receptor
EEKT: Expert-experience embedded knowledge transfer learning
Faster R-CNN: Faster regions with convolutional neural networks
GANs: Generative adversarial networks
GBM: Gradient boosting machine
GCN: Graph convolutional network
GoogLeNet: Google’s inception network
HCC: Hepatocellular carcinoma
H&E: Hematoxylin and eosin
HIPT: Hierarchical image pyramid transformer
HiPS: Histomic prognostic signature
HNSCC: Head and neck squamous cell carcinoma
IHC: Immunohistochemistry
KNN: K-nearest neighbor
LAION: Large-scale artificial intelligence open network
LC25000: Lung and colon cancer histopathological images
LDA: Linear discriminant analysis
LLaVA: Large language and vision assistant
LNM: Lymph node metastasis
LVI: Lymphovascular invasion
LYON19: Lymphocyte detection
mIF: Multiplex immunofluorescence
MIL: Multiple instance learning
MI-Zero: Multiple instance zero-shot transfer for histopathology images
ML: Machine learning
MoNuSeg: Multi-organ nucleus segmentation
MoNuSAC: Multi-organ nuclei segmentation and classification challenge
mSTAR: Multimodal self-taught pre-training
MSKCC: Memorial sloan kettering cancer center
MSI: Microsatellite instability
MVI: Microvascular invasion
NAC: Neoadjuvant chemotherapy
NB: Naïve bayes
NeuLy-IHC: Neutrophil lymphocyte and immunohistochemistry dataset
NSCLC: Non-small cell lung cancer
NuInsSeg: Nuclei instance segmentation
PAIP: Pathology artificial intelligence platform
PCam: PatchCamelyon
PLIP: Pathology language-image pre-training
QDA: Quadratic discriminant analysis
R-CNN: Region-based CNN
RCC: Renal cell carcinoma
ResNet: Residual network
RF: Random forest
RNN: Recurrent neural network
ROI: Region of interest
SigLIP: Sigmoid loss for language-image pre-training
SVM: Support vector machine
TABS: Tumor plus adjacent benign signature
TCGA: The Cancer Genome Atlas
TIL: Tumor-infiltrating lymphocyte
TITAN: Transformer-based pathology image and text alignment network
TME: Tumor microenvironment
TNBC: Triple-negative breast cancer
TSR: Tumor-stroma ratio
TUPAC16: Tumor proliferation assessment grand challenge 16
U-Net: U-shaped network
VAE: Variational auto-encoders
ViT: Vision transformer
WHO: World Health Organization
WSI: Whole-slide image
