# Supplementary material for: Artificial intelligence in digital pathology diagnosis and analysis: technologies, challenges, and future prospects
Source: Mil Med Res. 2026 Jan 4;12:93. doi: 10.1186/s40779-025-00680-6 (PMC12765299; doi:10.1186/s40779-025-00680-6)
Supplement: Supplementary file 1 — Additional file1. Table S1 Comparison of pathological large models on classification tasks across multiple cancer datasets. Table S2 Comparison of pathological large models on prognostic tasks across cancer datasets. [file 40779_2025_680_MOESM1_ESM.pdf]

**Table S1** Comparison of pathological large models on classification tasks across multiple cancer datasets

| Method | Cancer type | Metric type | AUC | Dataset |
|---|---|---|---|---|
| HIPT | BRCA | AUC | 0.830 | TCGA-BRCA |
| | PRAD | AUC | 0.768 | PANDA (1000 slides) |
| | LUAD | AUC | 0.952 | TCGA-LUAD |
| | RCC | AUC | 0.980 | TCGA-RCC |
| | CRC | AUC | 0.903 | TCGA CRC-MSI |
| CTransPath | BRCA | AUC | 0.544 | TCGA-BRCA |
| | PRAD | AUC | 0.684 | PANDA (1000 slides) |
| | LUAD | AUC | 0.973 | TCGA-NSCLC |
| | RCC | AUC | 0.991 | TCGA-RCC |
| | CRC | AUC | 0.887 | TCGA CRC-MSI |
| UNI | BRCA | AUC | 0.721 | TCGA-BRCA |
| | PRAD | AUC | 0.582 | PANDA (1000 slides) |
| | LUAD | AUC | 0.955 | TCGA-NSCLC |
| | RCC | AUC | 0.976 | TCGA-RCC |
| | CRC | AUC | 0.890 | TCGA CRC-MSI |
| CHIEF | BRCA | AUC | 0.912 | TCGA-BRCA |
| | PRAD | AUC | 0.854 | PANDA (1000 slides) |
| | LUAD | AUC | 0.982 | TCGA-LUAD |
| | RCC | AUC | 0.981 | TCGA-RCC |
| | CRC | AUC | 0.904 | TCGA CRC-MSI |
| CONCH | BRCA | AUC | 0.705 | TCGA-BRCA |
| | PRAD | AUC | 0.722 | PANDA (1000 slides) |
| | LUAD | AUC | 0.975 | TCGA-LUAD |
| | RCC | AUC | 0.975 | TCGA-RCC |
| | CRC | AUC | 0.850 | TCGA CRC-MSI |
| TITAN | BRCA | AUC | 0.884 | TCGA-BRCA |
| | PRAD | AUC | 0.823 | PANDA (1000 slides) |
| | LUAD | AUC | 0.960 | TCGA-LUAD |
| | RCC | AUC | 0.969 | TCGA-RCC |
| | CRC | AUC | 0.892 | TCGA CRC-MSI |

*HIPT* hierarchical image pyramid transformer, *CHIEF* clinical histopathology imaging evaluation foundation, *CONCH* contrastive learning from captions for histopathology, *TITAN* transformer-based pathology image and text alignment network, UNI towards a general-purpose foundation model for computational pathology, TCGA the cancer genome atlas, *BRCA* breast cancer, *PRAD* prostate adenocarcinoma, *LUAD* lung adenocarcinoma, *RCC* renal cell carcinoma, *CRC* colorectal cancer, *MSI* microsatellite instability

**Table S2** Comparison of pathological large models on prognostic tasks across cancer datasets

| Method | Cancer type | Metric type | C-index | Dataset |
|---|---|---|---|---|
| HIPT | LIHC | C-index | 0.557 | TCGA-LIHC |
| | LUAD | C-index | 0.538 | TCGA-LUAD |
| CTransPath | LIHC | C-index | 0.574 | TCGA-LIHC |
| | LUAD | C-index | 0.553 | TCGA-LUAD |
| UNI | LIHC | C-index | 0.572 | TCGA-LIHC |
| | LUAD | C-index | 0.558 | TCGA-LUAD |
| CHIEF | LIHC | C-index | 0.627 | TCGA-LIHC |
| | LUAD | C-index | 0.640 | TCGA-LUAD |
| CONCH | LIHC | C-index | 0.608 | TCGA-LIHC |
| | LUAD | C-index | 0.653 | TCGA-LUAD |
| TITAN | LIHC | C-index | 0.578 | TCGA-LIHC |
| | LUAD | C-index | 0.585 | TCGA-LUAD |

Performance was evaluated using the C-index. *HIPT* hierarchical image pyramid transformer, *CHIEF* clinical histopathology imaging evaluation foundation, *CONCH* contrastive learning from captions for histopathology, *TITAN* transformer-based pathology image and text alignment network, *UNI* towards a general-purpose foundation model for computational pathology, *TCGA* the cancer genome atlas, *LIHC* liver hepatocellular carcinoma, *BRCA* breast cancer, *LUAD* lung adenocarcinoma
